# Effects of Epigenetic Modification and High Hydrostatic Pressure on Polyketide Synthase Genes and Secondary Metabolites of *Alternaria alternata* Derived from the Mariana Trench Sediments

**DOI:** 10.3390/md21110585

**Published:** 2023-11-10

**Authors:** Qingqing Peng, Yongqi Li, Jiasong Fang, Xi Yu

**Affiliations:** Shanghai Engineering Research Center of Hadal Science and Technology, College of Marine Sciences, Shanghai Ocean University, Shanghai 201306, China; d220200047@st.shou.edu.cn (Q.P.);

**Keywords:** Mariana Trench, fungi, epigenetic modification, polyketide genes, secondary metabolites, one strain many compounds, high hydrostatic pressure

## Abstract

The hadal biosphere is the most mysterious ecosystem on the planet, located in a unique and extreme environment on Earth. To adapt to extreme environmental conditions, hadal microorganisms evolve special strategies and metabolisms to survive and reproduce. However, the secondary metabolites of the hadal microorganisms are poorly understood. In this study, we focused on the isolation and characterization of hadal fungi, screening the potential strains with bioactive natural products. The isolates obtained were detected further for the polyketide synthase (PKS) genes. Two isolates of *Alternaria alternata* were picked up as the representatives, which had the potential to synthesize active natural products. The epigenetic modifiers were used for the two *A. alternata* isolates to stimulate functional gene expression in hadal fungi under laboratory conditions. The results showed that the chemical epigenetic modifier, 5-Azacytidine (5-Aza), affected the phenotype, PKS gene expression, production of secondary metabolites, and antimicrobial activity of the hadal fungus *A. alternata*. The influence of epigenetic modification on natural products was strongest when the concentration of 5-Aza was 50 μM. Furthermore, the modification of epigenetic agents on hadal fungi under high hydrostatic pressure (HHP) of 40 MPa displayed significant effects on PKS gene expression, and also activated the production of new compounds. Our study demonstrates the high biosynthetic potential of cultivable hadal fungi, but also provides evidence for the utility of chemical epigenetic modifiers on active natural products from hadal fungi, providing new ideas for the development and exploitation of microbial resources in extreme environments.

## 1. Introduction

The ocean covers 71% of the surface of the Earth, making it the largest biosphere on this planet. The average depth of the ocean is 3800 m. Depths below 1000 m are known as the deep sea, while depths below 6000 m are classified as the hadal zone [1]. The hadal region is the most enigmatic ecosystem on our planet, representing an unparalleled extreme deep-sea environment on Earth. It is characterized by continuous darkness (no light), low temperature (typically about 3 °C), high hydrostatic pressure (up to 110 MPa) [2], poor food resources, isolated and complex terrain, and high seismic activity [3,4,5,6]. The hadal extreme environmental characteristics can facilitate the formation of a unique material circulation and energy flow system in the hadal environment [7]. Under extreme environmental conditions, marine microorganisms evolve distinctive metabolites to survive and proliferate [8]. Studies have reported that deep-sea microorganisms possess the potential to yield unprecedented compounds with antibacterial, anti-tumor, antiprotease, and antiviral activities [9]. In addition, among the marine microorganisms with bioactivities, marine fungi are considered to be the most promising source of sustainable drug utilization and an important source of useful secondary metabolites in drug discovery [10], due to their diverse structure and activity of secondary metabolites, high innovation index, strong drug formation, and high yield [11].

Polyketides are a class of natural secondary metabolites produced by bacteria, actinomyces, fungi, and plants. These natural products have potent biological activities of anti-infection, anti-fungi, anti-tumor, and anti-oxidative, being one of the most important sources of lead compounds in drug discovery. Polyketide compounds are mainly produced by polyketide synthase (PKS) gene clusters and catalyzed by PKSs at the first step [12], which can be classified into type I, II, and III, depending on the structure and reaction mechanism. Type I PKSs produce a vast array of biomedically important secondary metabolites, such as the antibiotic erythromycin, and immunosuppressant or antiparasitic ivermectin derivatives [13]. Numerous novel polyketide compounds have been found and reported to have biological and ecological functions in marine microbes. Therefore, the PKS genes were employed to evaluate the potential of fungi to produce natural products as a functional gene-based molecular screening approach.

Fungi are hosts for many novel biosynthetic gene clusters (BGCs) [14]. Changes in biotic and abiotic conditions in the natural environment trigger the silencing and expression of biosynthetic gene clusters of secondary metabolites in the fungal chromatin. Essentially, the triggers are the sum of environmental and nutrient factors that have a significant impact on the production of fungal secondary metabolites [15]. However, artificially prepared media in the laboratory are often poor substitutes for fungal fermentation conditions, resulting in most BGCs not producing detectable concentrations of metabolites when cultured under traditional laboratory conditions and being considered “silent” or “cryptic” [16,17,18]. Therefore, traditional fungi culture methods have been unable to indicate more compounds with novel structures and functions from fungi. Fortunately, some of the strategies used to awaken “silent” genes have been shown to have a significant effect on the production of secondary metabolites [19]. In 2008, Williams initially reported that epigenetic modifiers were effective tools for rationally controlling the native expression of fungal biosynthetic pathways, consequently generating new biomolecules [20]. Methods of activating fungal culture with chemical epigenetic modifiers have been proven to be a simple and effective way to find secondary metabolites with novel structures in fungi [21]. The main functions of chemical epigenetic modifiers, namely, histone deacetylase (HDAC) and DNA methyl transferase (DNMT), are to interfere with the production of fungal secondary metabolites and activate the expression of silencing biosynthetic pathways [22]. Currently, it has become a focus of research and has broad prospects for using chemical epigenetic modifiers to activate silent expression pathways to discover novel structures, new bioactive compounds, and in-depth exploration of the fungal metabolic potential. However, the existing studies have not involved hadal fungi.

Due to the obstacles of extremely deep water and high hydrostatic pressure (HHP), the techniques to obtain hadal samples in situ and cultivate hadal microorganisms are severely restricted, especially in the research field of fungi. Therefore, most research on natural bioactive compounds from marine fungi has been conducted in atmospheric pressure cultivation environments. The marine fungal secondary metabolites that have been reported were also produced under atmospheric conditions [23,24]. Unfortunately, there were insufficient technology and precise data to adequately describe the metabolic mechanism and function of hadal fungi. The unique biosynthetic pathway of secondary metabolites under HHP conditions was also unknown. This hinders the investigation of hadal life processes and restricts the utilization of these valuable microbial resources. However, the HHP serves as one of the extreme characteristics of the survival environment for hadal fungi, making the study of hadal fungi highly significant. In addition, Zhao [25] demonstrated that fungal strains from different environments possess unique structures and groups of natural products. Therefore, the exploration of the contribution to human production and life of hadal fungi has very broad prospects of development.

In this study, the culture experiment was conducted to expand the existing database of fungal strains by isolating hadal fungi under laboratory conditions, while the obtained isolates were screened for the PKS gene to evaluate the medicinal potential of hadal fungi. Meanwhile, we cultured the representative of hadal-derived fungi, *Alternaria alternata*, with epigenetic modifiers (5-Aza) under laboratory conditions and detected changes in fungal phenotypes, PKS gene expression, secondary metabolites, and bioactivity to explore the effects of chemical epigenetic modifiers on hadal fungi. The hadal fungi were reintroduced to the HHP environment and cultured with epigenetic modifier stimulation to observe changes in PKS gene expression, secondary metabolites, and bioactivity. The aim is to investigate whether epigenetic stimulation is also effective in the HHP environment, and whether the combination of both stimulation modes (HHP and epigenetic modifiers) could yield novel natural compounds. Based on the limited research on fungi in hadal environments, our research has significant theoretical and practical implications and also provides new ideas for the development and exploitation of microbial resources in the deepest oceans.

## 2. Results

### 2.1. Isolation and Identification of Hadal Sediment-Derived Fungi

**Figure 1:**
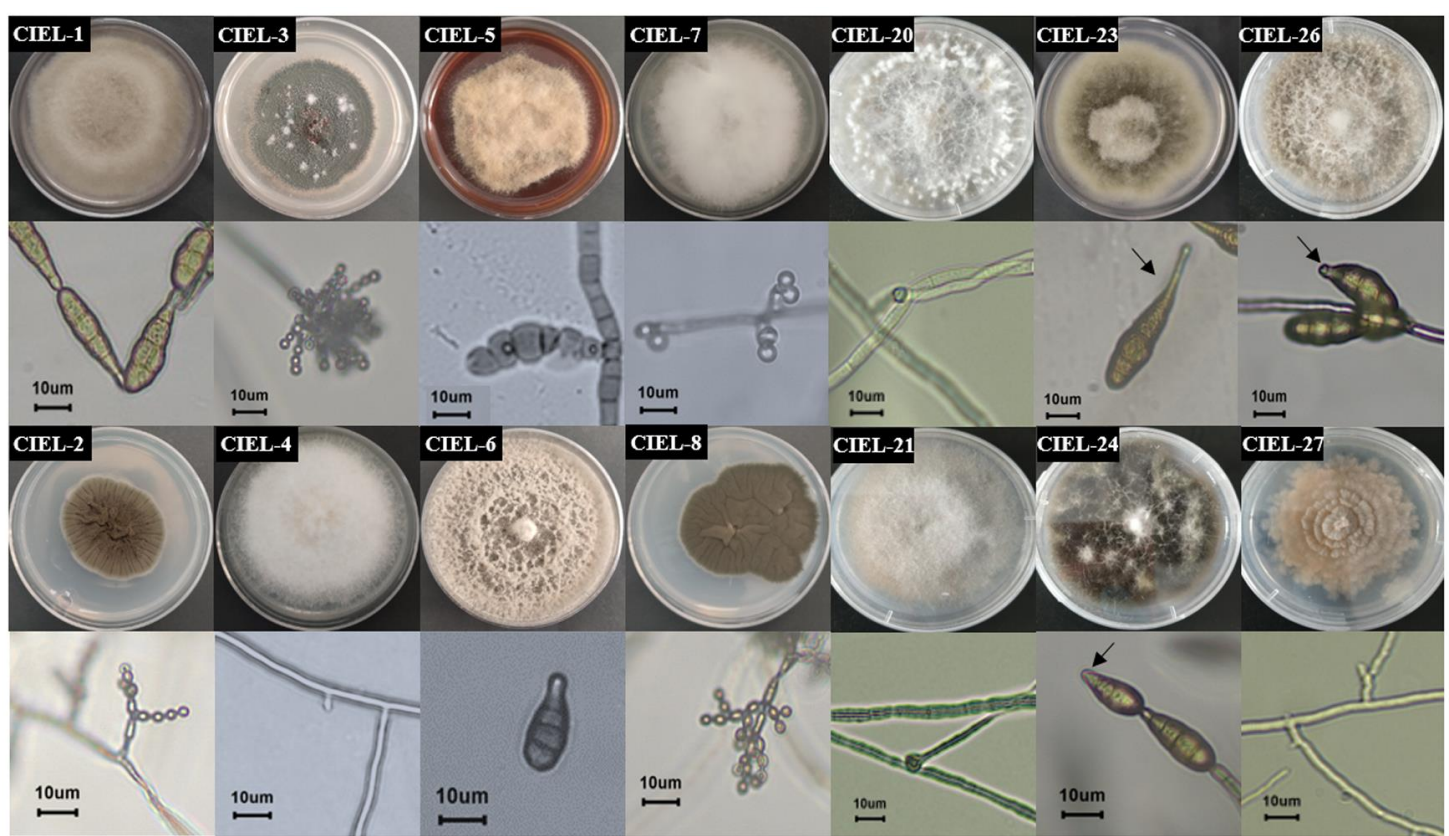
Colony phenotypes of 14 strains of fungi isolated from the sediments of the Mariana Trench. Colony phenotypes, mycelium, and spores under ×40 microscope of 14 strains were obtained by culturing PDA for 10 days at 28 °C. The arrow was used to mark the spore beaks. The scale was 10 μm.

In this study, six fungal strains, including two from 6477 m and four from 7332 m, were isolated from the hadal sediment of the Mariana Trench, expanding the existing database of hadal fungal species. Based on morphological identification and ITS sequence analysis (ITS1/ITS4), the isolated strains were identified and named *Arthrinium* sp. CIEL 20 (CIEL 20), *Didymella* sp. CIEL 21 (CIEL 21), *Alternaria alternata* CIEL 23 (CIEL 23), *Alternaria alternata* CIEL 24 (CIEL 24), *Alternaria alternata* CIEL 26 (CIEL 26), and *Preussia* sp. CIEL 27 (CIEL 27) (Figure 1, Table S1). After being cultured on PDA for 10 days at 28 °C, the morphological characteristics of six hadal-derived fungi were recorded. Similar to that described by Kwon [26], the morphological characteristics of *Arthrinium* sp. CIEL 20 showed white colonies, which grow rapidly with uneven surfaces and regular margins, white and highly branched mycelia, and spherical or ellipsoidal spores (Figure 1). The colony morphology of *Didymella* sp. CIEL 21 showed pale pink colonies with flat surfaces and regular margins, colorless and multi-branched mycelia, and spherical or ellipsoidal spores (Figure 1). The characteristics of *A. alternata* CIEL 23, *A. alternata* CIEL 24, and *A. alternata* CIEL 26 displayed the typical characteristics of *Alternaria* sp. (microbiologyinfo.com), including rapidly growing gray to olive or olive-brown colonies, brown downy and multibranched mycelia, and olive-green to brown single or chain-forming conspicuous septate spores. *A. alternata* CIEL 23 exhibited colonies with regular margins of intermediate gray-edged olive green to gray, gray downy, and single-chain spores with long beaks. *A. alternata* CIEL 24 showed olive-green colonies with irregular (wavy) margins, and single-chain spores with short beaks. *A. alternata* CIEL 26 showed colonies with regular margins of intermediate white with gray, colorless hyphae, and brown multi-chain spores with short beaks (Figure 1). The morphology of the colony of *Preussia* sp. CIEL 27 showed white to light cream sectors, ascomata scattered or aggregated, superficial or partially immersed, margin irregular, and no observed sporulation after being cultured in PDA for 10 days at 28 °C (Figure 1).

**Figure 2:**
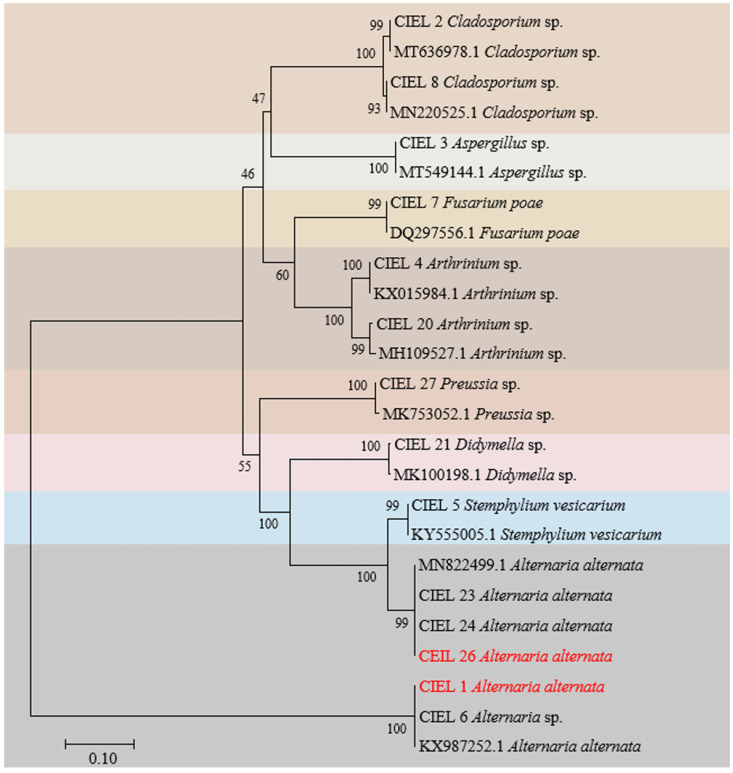
Neighbor-joining phylogenetic tree based on ITS sequences (ITS1/ITS4 primers) of the 14 fungi isolated from sediments of the Mariana Trench. Numbers at branches indicate bootstrap values of neighbor-joining analysis from 1000 replications. The scale bar represents 0.10 nucleotide substitutions per site. Different species were represented by different colored areas.

Preliminary analysis showed that the six isolates obtained here and the eight previously reported fungi [27] shared the same sample origin (Table S1), and all possessed potential bioactivities (Table S2). Next, we focused on these 14 strains of hadal-derived fungi to explore the chemical diversity and biosynthetic potential of hadal fungi in laboratory conditions. Phylogenetic analysis of the ITS sequence showed that all the fungi isolated from the hadal sediment samples originated closely to terrestrial plant pathogenic fungi in the NCBI database at high similarity (100%) (Figure 2), which supports the hypothesis that marine sources of fungi are likely to be terrestrial “wash-ins” [28].

### 2.2. Screening of the PKS Genes in Hadal-Derived Fungi

**Figure 3:**
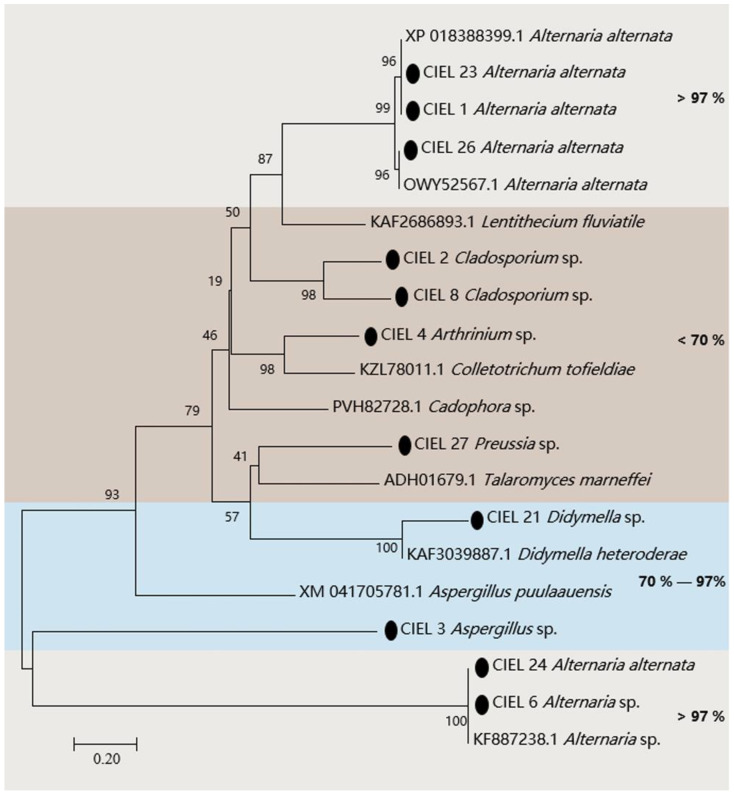
Neighbor-joining phylogenetic tree based on PKS sequences of the 11 isolates from sediments of the Mariana Trench. Numbers at branches indicate bootstrap values of neighbor-joining analysis from 1000 replications. The scale bar represents 0.20 nucleotide substitutions per site. The black spot represents nucleotide sequences obtained in this study. Different homologies were represented by different colored areas.

The presence of the PKS gene in fungi is one of the clues to assess whether the fungi have the ability to produce polyketides. Hence, we assessed the biosynthetic potential of the hadal fungi by screening for PKS genes in the hadal-derived fungi. PKS genes (ca. 800 bp) of 11 strains of fungi were detected in 14 isolates by KAF1/KAR1 (5′-GARKSICAYGGIACIGGIAC/5′-CCAYTGIGCICCRTGICCIGARAA) primers. The obtained PKS gene sequences were compared by BlastX and the phylogenetic tree was constructed (Figure 3). The results demonstrated that the four strains (*A. alternata* CIEL 1, *A. alternata* CIEL 23, *A. alternata* CIEL 24, and *A. alternata* CIEL 26) showed more than 98% similarity with the polyketide synthase PksJ of *Alternaria alternata*, which was involved in the biosynthesis of polyketide signaling metabolites [29] (Table S3). Among them, the two strains (*A. alternata* CIEL 1 and *A. alternata* CIEL 23) had identical PKS protein sequences of 98.58% similarity with that of *Alternaria alternata* (XP 018388399.1), suggesting that they might share a conserved PKS origin. Another PKS protein of *Alternaria* sp. CIEL 6 showed 97.75% similarity with the PKS gene partial CDS sequence of *Alternaria* sp. (KF887238.1). In contrast, some of the isolates, such as *Aspergillus* sp. CIEL 3 and *Didymella* sp. CIEL 21, had a relatively lower homology (70–97%) to annotated type I PKS protein sequences on NCBI, indicating their genetic potential for the synthesis of polyketides. In addition, the PKS protein sequences determined in some strains (*Cladosporium* sp. CIEL 2, *Arthrinium* sp. CIEL 4, *Cladosporium* sp. CIEL 8, and *Preussia* sp. CIEL 27) have less than 50% homology with that of known strains in NCBI, which may be potentially novel as undescribed fungal PKSs (Figure 3). The phylogenetic analysis of protein fragments indicated that most isolated hadal fungi harbor PKSs, indicating their biosynthetic potentials.

### 2.3. Chemical Epigenetic Modifier Affects Fungal Phenotype

Epigenetic modifications alter chromosomal DNA and histones, not the primary DNA sequence. They affect specific sites or chromosomes without altering the DNA sequence, causing heritable phenotype changes [29]. To activate biosynthetic genes in hadal fungi, we determined the effects of two epigenetic modifiers on colony phenotypes and metabolite production in different media for 10 days at 28 °C. In our study (Figure S1), it can be concluded that the 5-Aza (1 mM) had a clearly visible effect on the phenotypic changes of the isolates, such as irregular mycelial globular projections appearing on the surface of the colony, increased aerial mycelia, and white mycelia. Furthermore, the minimum inhibitory concentration (MIC) results showed that SAHA, 5-Aza, and DMSO all affected the production of secondary metabolites of the isolates (Table S2).

**Figure 4:**
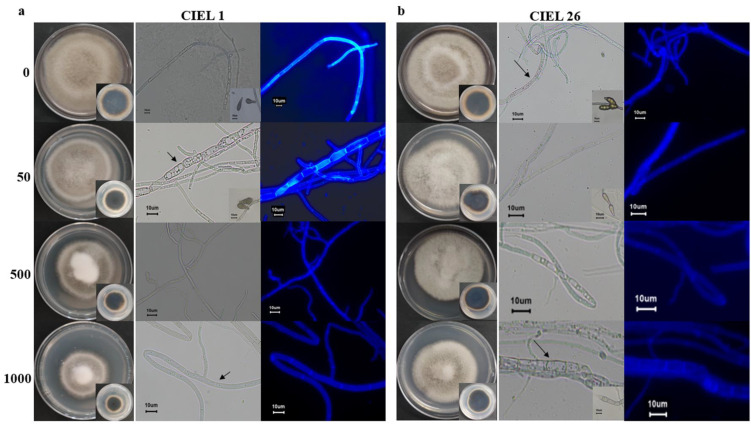
Phenotypes of two strains of *A. alternata* cultured with epigenetic modification at different concentrations. Under the same conditions, two species of *A. alternata* were cultured with four concentrations of 5-Aza (0, 50 μM, 500 μM, and 1000 μM) on PDA at 28 °C for 10 days: (**a**) Shows the colony phenotypes, the pigmentation on the back of the colonies, the mycelia and spore phenotypes taken under a ×40 microscope with a scale of 10 μm of *A. alternata* CIEL 1. (**b**) Shows the colony phenotypes, the pigmentation on the back of the colonies, the mycelia and spore phenotypes taken under a ×40 microscope with a scale of 10 μm of *A. alternata* CIEL 26. Among them, *A. alternata* CIEL 1 and *A. alternata* CIEL 26 had swollen mycelium (arrow).

Considering the influence of the agents on fungal phenotype and metabolites, we finally chose 5-Aza as the chemical epigenetic modifier and sterile water as the solvent for the preparation of the culture medium (Table S4). Based on the phenotypic changes and PKS analyses, two strains of *A. alternata* fungi (*A. alternata* CIEL 1 and *A. alternata* CIEL 26) were selected as target strains for further studies on the effects of chemical epigenetic modifiers on hadal fungi. The results of 5-Aza on the target strains showed that different concentrations of the epigenetic modifier had different effects on the growth rate, phenotype, pigmentation, and sporulation of *A. alternata* (Figure 4). Under the same culture conditions (cultured on PDA at 28 °C for 10 days), the effects of 5-Aza on *A. alternata* CIEL 1 and *A. alternata* CIEL 26 displayed a similar trend: the area of the colony decreased with increasing 5-Aza concentration, the mycelia of the strains swelled when the 5-Aza concentration was 50 μM, and the strains did not produce spores when the 5-Aza concentration was greater than 50 μM. However, when the 5-Aza concentration was 50 μM, only *A. alternata* CIEL 1 showed a significant increase in the number of vesicles in the mycelia (Figure 4a). After being cultured on PDA medium for 10 days at 28 °C, the spores of *A. alternata* CIEL 26 could only be observed at a 5-Aza concentration of 0 and 50 μM (Figure 4b).

### 2.4. Chemical Epigenetic Modifier Affects PKS Gene Expression in Fungi

Total RNA was extracted from fresh mycelia cultured under different concentrations of 5-Aza in SDB medium at 28 °C for 10 days on a rotating shaker (160 rpm). The qRT-PCR primers located in the KS structural domain were designed based on the sequences (530–832 bp) with the highest similarity (99.46%) of the PKS gene amplified by the degenerate primers. In order to select primers with significant amplification efficiency for the PKS genes and internal reference genes of *Alternaria* sp., the designed qRT-PCR primers and internal reference genes primers were validated. The results showed that primers AltqpksF1/AltqpksR1 (5′-GAAAGCGTCACCCTGAAGTA/5′-AAAGGAGGCAGTGGAGCA) had high amplification efficiency (1.82–1.94%) for all four strains of *Alternaria* sp., and primers ALTqG1F4/ALTqG1R4 (5′-GGCAAGACCATCCGTTTC/5′-CAGCAGAGGGAGCAGAAAT) were stably expressed (1.83–1.94%) in two strains of *A. alternata*.

**Figure 5:**
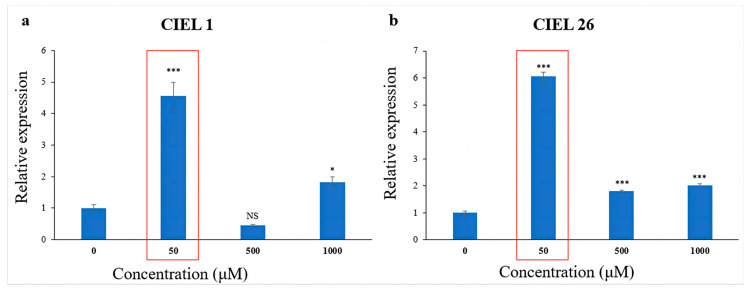
Statistical diagram of the relative expression of the PKS gene. Two strains of *A. alternata* were cultured with different concentrations of 5-Aza in SDB at 28 °C for 10 days. Blank control was set without the addition of 5-Aza. In the figure, (**a**) represents *A. alternata* CIEL 1, and (**b**) represents *A. alternata* CIEL 26. Compared with the blank control, results were considered significant at the level of P (NS *p* > 0.05, * *p* < 0.05, *** *p* < 0.001). When the concentration of 5-Aza was at 50 μM, the PKS expression of CIEL 1 and CIEL 26 was significantly increased (red box).

The effect of 5-Aza on the expression level of the PKS gene was detected using qRT-PCR. The results showed that the chemical epigenetic modifier (5-Aza) had different effects on PKS gene expression levels depending on concentration and fungal strains. Meanwhile, consistently, the expression levels of the PKS genes of the two *A. alternata* were significantly increased at 5-Aza concentrations of 50 μM (Figure 5). Compared with the blank control, the expression of the PKS gene increased 3.5-fold in *A. alternata* CIEL 1 (Figure 5a), and 5.0-fold in *A. alternata* CIEL 26 (Figure 5b) under 50 μM of 5-Aza in culture.

### 2.5. Chemical Epigenetic Modifier Affects the Components of Fungal Secondary Metabolites

**Figure 6:**
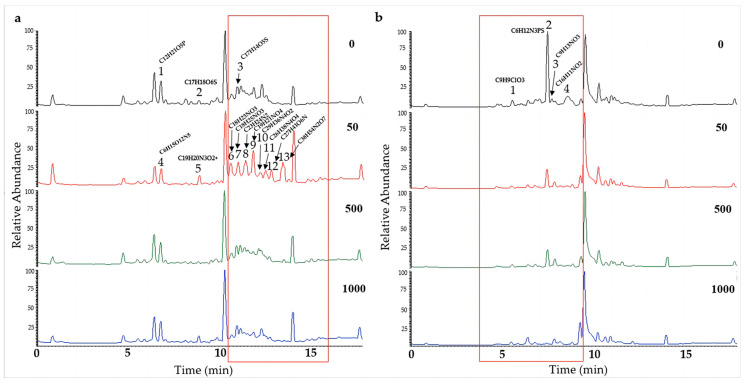
UPLC-MS/MS diagram of secondary metabolites of *A. alternata*. Two strains of *A. alternata* were cultured with different concentrations of 5-Aza in SDB at 28 °C for 10 days. In the figure, (**a**) represents *A. alternata* CIEL 1, and (**b**) represents *A. alternata* CIEL 26. The *X*-axis was the retention time (min), and the *Y*-axis was the relative response (%). The TIC of the products produced by the target strains in media containing different concentrations of 5-Aza (the number in the upper right corner of the picture indicates the concentration of 5-Aza) was indicated by different colored lines (black-0 μM, red-50 μM, green-1000 μM, and blue-1000 μM). The different signal peaks were marked in the red box.

To further determine the effect of chemical epigenetic modifiers on the natural products of hadal fungi, the extracts of the two strains of *Alternaria alternata* (*A. alternata* CIEL 1 and *A. alternata* CIEL 26), cultured on SDB with different concentrations of 5-Aza at 28 °C for 10 days at 160 rpm, were profiled by UPLC-MS/MS. In the comparison of metabolites from the same strains under different concentrations of 5-Aza, different peaks in the total ion chromatogram (TIC) were observed. The obtained mass spectrum data were blasted in the COCONUT database, the NP Atlas database, and the StreptomeDB database to find the corresponding compounds. In our study, we found that the production of secondary metabolites changed with the addition of epigenetic agents (Figure 6 and Figure S2). A significant alteration in signal peaks was observed in *A. alternata* CIEL 1 (Figure 6a) at a concentration of 50 μM (5-Aza) compared to the blank control. Three new compound signals A1-4 (6.81 min), A1-5 (8.91 min), A1-7 (11.04 min), and seven new peaks, A1-6 (10.63 min), A1-8 (11.44 min), A1-9 (11.88 min), A1-10 (12.26 min), A1-11 (12.54 min), A1-12 (12.84 min), and A1-13 (13.50 min) contributed to the observed changes. Through blasting in the database, the compounds were identified putatively as A1-1 (C_12_H_21_O_5_P), A1-2 (C_17_H_18_O_6_S), A1-3 (C_23_H_24_N_2_), A1-4 (C_6_H_15_O_12_N_5_), A1-5 (C_19_H_20_N_3_O_2_+), A1-6 (C_18_H_23_NO_3_), A1-7 (C_18_H_25_NO_3_), A1-8 (C_23_H_24_N_2_), A1-9 (C_19_H_21_NO_4_), A1-10 (C_29_H_36_N_4_O_2_), A1-11 (C_26_H_38_N_4_O_4_), A1-12 (C_27_H_41_O_6_N), and A1-13 (C_38_H_54_N_2_O_7_). Moreover, compounds A1-1 and A1-4, A1-2 and A1-5, and A1-3 and A1-7 share the same RT; however, their compositions differ. In addition, the TIC of extract of *A. alternata* CIEL 26 also showed changes with the addition of 5-Aza (Figure 6b). Four peaks, specifically A26-1 (C_9_H_9_ClO_3_, 5.57 min), A26-2 (C_6_H_12_N_3_PS, 7.51 min), A26-3 (C_9_H_13_NO_3_, 7.75 min), and A26-4 (C_16_H_11_NO_2_, 8.60 min), underwent changes in the TIC of *A. alternata* CIEL 26 after the addition of 5-Aza. Our results indicated that chemical epigenetic modifiers impacted the biosynthesis of those natural compounds in hadal fungi.

### 2.6. Chemical Epigenetic Modifier Affects Antimicrobial Activity

**Figure 7:**
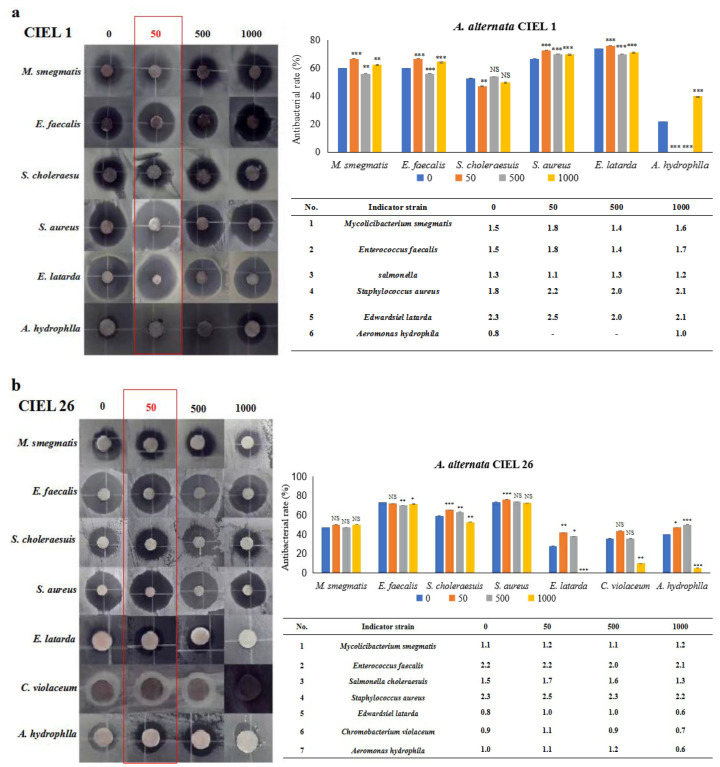
Statistical diagram of the secondary metabolite activities of *A. alternata*. Two strains of *A. alternata* were cultured with different concentrations of 5-Aza on SDB at 28 °C for 10 days. In the figure, (**a**) represents *A. alternata* CIEL 1, and (**b**) represents *A. alternata* CIEL 26. The left shows the results of the inhibitory zone, and the right shows the inhibitory rate calculated according to the diameter of the inhibitory zone. Error bars indicate standard deviation. Compared with 0, results were considered to be significant at the level of *p* (NS *p* > 0.05, * *p* < 0.05, ** *p* < 0.01, *** *p* < 0.001). The part of the antibacterial zone that most increased was marked in the red box.

Next, six strains of human pathogens (*Staphylococcus aureus* ATCC25923, *Enterococcus faecalis* FA2-2, *Escherichia coli* MG1655, *Chromobacterium violaceum* ATCC12472 CV026, *Salmonella choleraesuis*, and *Mycobacterium smegmatis*) and three strains of aquatic pathogens (*Edwardsiel latarda*, *Klebsiella pnenmoniae*, and *Aeromonas hydrophlla*) were chosen to conduct the antibacterial activity assay by the Kirby–Bauer method. Our results demonstrated that epigenetic modifiers (5-Aza) had an influence on the antibacterial activity of secondary metabolites of *A. alternata*. Furthermore, the regulated effects varied within the strains but were consistent with the strongest effect at 5-Aza concentrations of 50 μM (Figure 7). For example, *A. alternata* CIEL 1 produced compounds that significantly inhibited the growth of six pathogens (*M. smegmatis*, *E. faecalis*, *S. choleraesuis*, *S. aureus*, *E. latarda*, and *A. hydrophlla*) after 10 days of cultivation at 28 °C in SDA medium. The inhibition rates were 60.0%, 60.0%, 52.6%, 66.7%, 73.9%, and 21.7% respectively. The addition of 50 μM of 5-Aza to CIEL 1 improved the antimicrobial efficacy against *M. smegmatis*, *E. faecalis*, *S. aureus*, and *E. latarda* by 6.7%, 6.7%, 6.1%, and 2.1%, respectively. However, it decreased the antimicrobial efficacy against *S. choleraesuis* and *A. hydrophlla* by 5.6% and 21.7%, respectively (Figure 7a). The extracts of *A. alternata* CIEL 26 exhibited considerable activity against seven pathogens (*M. smegmatis*, *E. faecalis*, *S. choleraesuis*, *S. aureus*, *E. latarda*, *C. violaceum*, and *A. hydrophlla*) with an inhibition rate of 47.1%, 73.1%, 59.1%, 73.5%, 28.0%, 35.7%, and 40.0%, respectively. When 50 μM of 5-Aza was introduced, the antibacterial activities of *A. alternata* CIEL 26 on six pathogens (*M. smegmatis*, *S. choleraesuis*, *S. aureus*, *E. latarda*, *C. violaceum*, and *A. hydrophlla*) were increased by 2.9%, 6.3%, 2.8%, 13.9%, 8.0%, and 7.1%, respectively, while the antimicrobial activity against *E. faecalis* was decreased by 1.3% (Figure 7b).

### 2.7. Chemical Epigenetic Modifier Affects PKS Gene Expression under HHP Conditions

**Figure 8:**
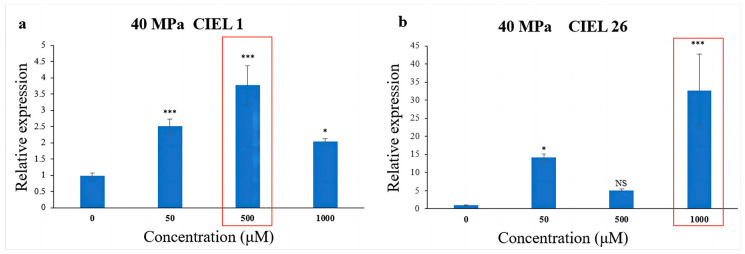
Statistical diagram of the relative expression of the PKS gene under the HHP conditions. With different concentrations of 5-Aza, two strains of *A. alternata* were cultured under the HHP (40 MPa) conditions for 14 days and then cultured in SDB at 28 °C for 10 days. Blank control was set without the addition of 5-Aza. In the figure, (**a**) represents *A. alternata* CIEL 1, and (**b**) represents *A. alternata* CIEL 26. Compared with the blank control, results were considered significant at the level of *p* (NS *p* > 0.05, * *p* < 0.05, *** *p* < 0.001). The concentrations of 5-Aza that showed the most increased PKS expression were highlighted.

To further understand the effect of epigenetic modification on biosynthetic genes under HHP, spore suspensions treated with HHP for 14 days were cultured with varying concentrations of 5-Aza. The expression of the PKS gene in *A. alternata* was detected by qRT-PCR. The results showed that the chemical epigenetic modifier (5-Aza) also affected the expression levels of the PKS gene under high-pressure (40 MPa) stimulation, but the degree of the effect varied in different strains (Figure 8). Compared with the blank control, the expression level of the PKS gene in *A. alternata* CIEL 1 increased by 2.8-fold when cultured with 500 μM 5-Aza in culture (Figure 8a). Whereas in *A. alternata* CIEL 26, the expression level in culture with 1000 μM of 5-Aza was 32.6 times higher than that with 0 μM 5-Aza (Figure 8b).

### 2.8. Chemical Epigenetic Modifier Affects the Components of Secondary Metabolites under HHP Conditions

**Figure 9:**
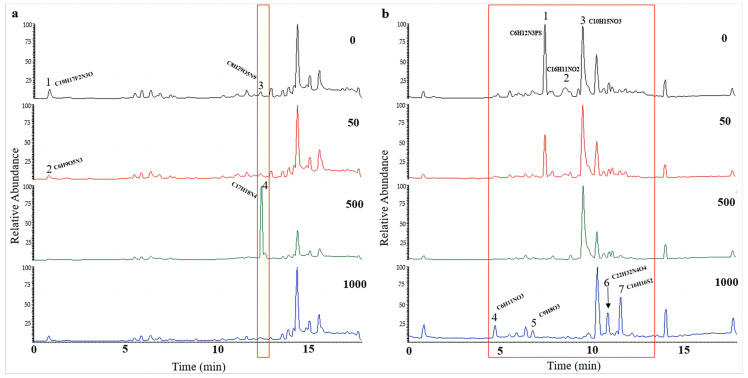
UPLC-MS/MS diagram of secondary metabolites of *A. alternata* under HHP conditions. With different concentrations of 5-Aza, two strains of *A. alternata* were cultured under the HHP (40 MPa) conditions for 14 days and then cultured in SDB at 28 °C for 10 days. In the figure, (**a**) represents *A. alternata* CIEL 1, and (**b**) represents *A. alternata* CIEL 26. The *X*-axis was the retention time (min), and the *Y*-axis was the relative response (%). The TIC of the products produced by the target strains in media containing different concentrations of 5-Aza (the number in the upper right corner of the picture indicates the concentration of 5-Aza) was indicated by different colored lines (black-0 μM, red-50 μM, green-1000 μM, and blue-1000 μM). The different signal peaks were marked in the red box.

UPLC-MS/MS was used to investigate the influence of 5-Aza on secondary metabolites of *A. alternata*. After being stimulated under HHP conditions for 14 days, the spores were cultured in SDB on a rotating shaker (160 rpm) for 10 days at atmospheric pressure. In our study, the impacts of epigenetic modification under HHP on the production of secondary metabolites also depended on the concentrations of 5-Aza (Figure 9 and Figure S3). For CIEL 1, the characterization of compounds A1-40-1 and A1-40-2, A1-40-3, and A1-40-4, with RT of 0.84 min and 12.42 min, respectively, were altered due to the different concentrations of 5-Aza (Figure 9a). These compounds were identified as A1-40-1 (C_19_H_17_F_2_N_3_O), A1-40-2 (C_6_H_9_O_5_N_3_), A1-40-3 (C_8_H_2_O_5_N_9_), and A1-40-4 (C_17_H_18_N_4_). Four new peaks, A26-40-4 (4.74 min), A26-40-5 (6.82 min), A26-40-6 (10.91 min), and A26-40-7 (11.63 min), were observed in the TIC of *A. alternata* CIEL 26 after adding 1000 μM of 5-Aza (Figure 9b). Through blasting in the database, the four compounds were identified as C_6_H_11_NO_3_ (A26-40-4), C_9_H_8_O_3_ (A26-40-5), C_22_H_32_N_4_O_4_ (A26-40-6), and C_16_H_16_S_2_ (A26-40-7), respectively.

### 2.9. Chemical Epigenetic Modifier Affects Antimicrobial Activity under HHP Conditions

**Figure 10:**
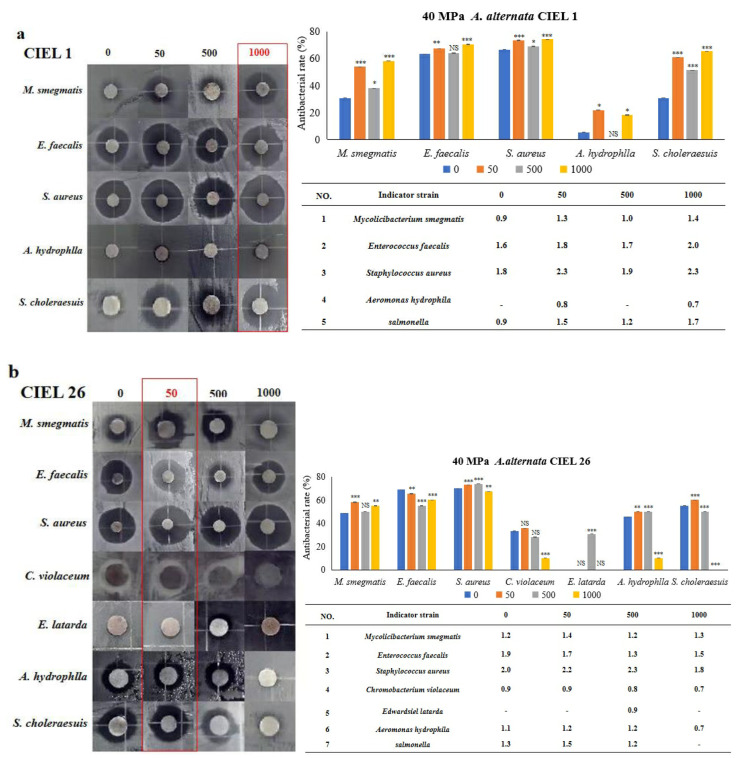
Statistical diagram of the secondary metabolite activities of *A. alternata* under the HHP conditions. With different concentrations of 5-Aza, two strains of *A. alternata* were cultured under the HHP (40 MPa) conditions for 14 days and then cultured in SDB at 28 °C for 10 days. In the figure, (**a**) represents *A. alternata* CIEL 1, and (**b**) represents *A. alternata* CIEL 26. The left shows the results of the inhibitory zone, and the right shows the inhibitory rate calculated according to the diameter of the inhibitory zone. Error bars indicate standard deviation. Compared with 0, results were considered to be significant at the level of *p* (NS *p* > 0.05, * *p* < 0.05, ** *p* < 0.01, *** *p* < 0.001). The part of the antibacterial zone that most increased was marked in the red box.

The Kirby–Bauer method was used to determine the effect of 5-Aza on the antimicrobial activity of secondary metabolites of *A. alternata*, which was stimulated under HHP conditions for 14 days and then cultured in SDB on a rotating shaker (160 rpm) for 10 days. Our results demonstrated that the inclusion of epigenetic modifiers (5-Aza) also induced changes in the antibacterial activity of secondary metabolites of *A. alternata* following cultivation under HHP conditions (Figure 10). For example, the secondary metabolites produced by *A. alternata* CIEL 1 were found to have antibacterial effects against five pathogens (*M. smegmatis*, *E. faecalis*, *S. aureus*, *A. hydrophlla*, and *S. choleraesuis*), with an inhibition rate of 30.8%, 63.3%, 66.7%, 5.3%, and 30.8%, respectively. When 1000 μM of 5-Aza was added, the antimicrobial activity against five pathogens (*M. smegmatis*, *E. faecalis*, *S. aureus*, *A. hydrophlla*, and *S. choleraesuis*) was enhanced, by 27.4%, 7.2%, 7.6%, 12.9%, and 34.6%, respectively (Figure 10a). The metabolites produced by *A. alternata* CIEL 26 demonstrated noteworthy impacts on six pathogens (*M. smegmatis*, *E. faecalis*, *S. aureus*, *C. violaceum*, *A. hydrophlla*, and *S. choleraesuis*) with inhibition rates of 48.6%, 69.0%, 70.0%, 33.3%, 45.5%, and 55.0%, respectively. The addition of 50 μM 5-Aza increased the antibacterial activities of five pathogens (*M. smegmatis*, *S. aureus*, *C. violaceum*, *A. hydrophlla*, and *S. choleraesuis*) by 9.6%, 3.1%, 2.4%, 4.5%, and 5.0%, respectively, but decreased the activity of *E. faecalis* by 3.6% (Figure 10b).

## 3. Discussion

Marine fungi account for more than 60% of the 456 newly reported marine microbial natural products [30]. This indicates that the unexploited scale of biosynthetic genes and bioactive compounds from marine fungi is huge and has great research importance. At present, remarkable progress has been made in the research of marine fungi in drug development, which produces chemical diversity agents with a wide range of antibacterial, antiviral, and anticancer properties in animal systems, emphasizing the importance of marine fungi as a source of medicinal natural products [31,32]. However, due to the specificity of the hadal environment, little research has been reported that currently involves hadal fungi. In our study, we isolated fungal strains from the Marian Trench sediments by the cultivable method, which enlarged the existing database of hadal fungal strains and provided the research basis for natural products research. Based on phylogenetic diversity analysis of the ITS sequence, all isolates from this study had 100% homology to terrestrial plant pathogenic fungi, suggesting that they might share a common ancestry. This result suggests that such strains might have their origin in the terrestrial environment [33], which raises intriguing ecological questions regarding the ability of terrestrial fungi to tolerate and adapt to marine conditions (pressure, temperature, and salinity). However, the majority of the culture conditions of hadal fungi were in the laboratory with ambient pressure, leading to the possibility of loss of expression of function genes. This knowledge can be exploited for applications such as activating silent gene clusters and thus enhancing the discovery of new natural products [34].

PKSs have been shown to synthesize natural products with diverse structures and biomedically important potentials [35]. At present, the research on marine fungi mainly focuses on genomics and metabolomics associated with PKS gene screening [36,37,38]. Few attempts have been made to detect the PKS genes in hadal fungi. Here, we screened the PKS genes of 14 hadal-derived fungi isolated from the Mariana Trench sediments. The results showed that the PKS genes were widespread in the hadal-derived fungi, which indicated their potential in the biosynthesis of polyketide compounds. Furthermore, the PKS genes were detected in the fungal strains with antimicrobial activities, for example, isolates *A. alternata* CIEL 1, *Aspergillus* sp. CIEL 3, *Arthrinium* sp. CIEL 4, *Alternaria* sp. CIEL 6, *Didymella* sp. CIEL 21, *A. alternata* CIEL 23, *A. alternata* CIEL 24, *A. alternata* CIEL 26, and *Preussia* sp. CIEL 27, implying their potential in the production of antimicrobial PKS compounds. However, some isolates that had not been detected for the PKS gene, for example, *S. vesicarium* CIEL 5, *F. poae* CIEL 7, and *Arthrinium* sp. CIEL 20, also showed antimicrobial activity, which may be due to the limit of primers to amplify their PKS sequences, or it may have resulted from other secondary metabolites instead of PKS compounds. In addition, the phylogenetic analysis showed that the three strains of *A. alternata* (*A. alternata* CIEL 1, *A. alternata* CIEL 23, and *A. alternata* CIEL 26) in this experiment had high similarity (>99.0%) of PKS gene sequences, which demonstrated that the PKS gene sequences are also highly conserved in hadal fungi. It was the first time to detect the diversity of PKS genes in hadal-derived fungi, which extended our knowledge of hadal fungi. However, the number of hadal fungi strains obtained was limited, which needs further investigation in the future.

Over the past two decades, a large number of compounds with significant biological activity have been produced by chemical epigenetic modifiers that stimulate silent gene expression in fungi [20,39,40]. Unfortunately, most studies on the effects of chemical epigenetic modifiers have focused on terrestrial and shallow marine fungi, with a few involving deep-sea fungi and almost none on hadal fungi. Long-term cultivation of hadal fungi under laboratory conditions causes the mutation of functional genes. In this study, we cultured hadal-derived fungi with an epigenetic modulator (5-Aza) under laboratory conditions, and demonstrated that chemical epigenetic modifiers also had an effect on the phenotype, PKS gene expression, secondary metabolite production, and antimicrobial activity of hadal-derived fungi. Poolchanuan found that the epigenetic modulator (valproic acid, VPA) can inhibit the production of polyketides by *Dothideomycete* sp., and a concentration of VPA greater than 300 μM can inhibit the growth of the fungus [41]. Our study’s findings were inconsistent with this outcome. We found that when the concentration of 5-Aza was 50 μM, the growth of *A. alternata* was significantly inhibited, and the effects of chemical epigenetic modifiers varied in strains. The promotion effect was strongest under the concentration of 5-Aza at 50 μM. 5-Aza is known to work by consuming enzyme activity to induce the demethylation and activation of silenced genes. We speculated that the homeostasis and self-protection mechanisms of organisms will be triggered due to the increase in epigenetic agents, which explains why the gene expression level increases significantly under the concentration of epigenetic modifiers (50 μM). The expression of the 5-Aza-stimulated silenced gene was slightly different in different organisms because of the difference in enzyme activity in different organisms. Furthermore, the existence of new compounds suggested that changing the traditional laboratory culture conditions (adding 5-Aza) could promote the expression of silenced genes or biosynthetic pathways. This result is consistent with the conclusion by Williams [20] that only a small fraction of the fungal-induced biosynthetic pathways encoding secondary metabolites can be expressed under laboratory culture conditions, which greatly limits the prospects for fungal biosynthesis, and with the fact that significantly fewer fungal active metabolites are known to be present in the clinic than fungal potential secondary metabolites. Although our result demonstrates the feasibility of using epigenetic modifiers to develop active natural products under laboratory culture conditions, after adding 5-Aza, the biological activities of extracts from *A. alternata* changed depending on the 5-Aza concentrations and species of pathogens. In other words, the active compounds produced by 5-Aza did not have wide antibacterial effects, and not all the compounds were activated by epigenetic regulation. To successfully activate silenced BGCs requires a proper understanding of the regulatory circuits controlling secondary metabolite gene clusters, but the signaling cues that trigger the expression of silenced or transiently expressed BGCs in the natural environment remain unclear [42].

Raghukumar and Damare [43,44] have analyzed the growth characteristics of deep-sea fungi under HHP (20 MPa) conditions and proved that deep-sea fungi are capable of growing normally under simulated deep-sea conditions. In a previous study, we demonstrated that HHP conditions were able to influence the biosynthesis of fungal secondary metabolites by regulating the expression of PKS genes [26,45]. It should be noted, however, that the silenced part of the gene was not taken into account. Epigenetic modifiers were used to stimulate the silencing genes. Therefore, we investigated the potential of combining HHP and epigenetic modifiers to generate unique compounds. Our study revealed that utilizing chemical epigenetic modifiers still impacted PKS gene expression, secondary metabolites, and bioactivities of the hadal fungi under HHP conditions. However, the results were different from those obtained with the use of epigenetic modifiers alone. A comparison of the UPLC-MS/MS results under atmospheric and high-pressure conditions revealed that new natural metabolites were also produced by hadal fungi in response to the combined effects of HHP and chemical epigenetic modifiers. Notably, the new natural compounds produced under HHP did not overlap with the compounds produced under atmospheric conditions. So, we speculated that the effects of pressure and epigenetic modifiers have the ability to cause hadal fungi to generate distinct compounds compared to those produced under normal pressure. The cause of this phenomenon is unknown and could potentially be attributed to the diverse adaptive and regulatory mechanisms of fungi inhabiting various hydrostatic environments. Therefore, researching these adaptive mechanisms is imperative, as it not only facilitates the development and exploitation of hadal compound resources but also holds significant theoretical implications for comprehending the survival and evolution of hadal organisms. Although exploring the effects of chemical epigenetic regulators on hadal fungi under extreme environmental conditions remains challenging, our study provides instructive evidence and takes an important step forward in improving the medical potential of hadal fungi.

## 4. Materials and Methods

### 4.1. Fungal Isolation and Purification

Hadal sediment samples were collected from the Mariana Trench (11°20′ N, 142°11.5′ E) at about 5437 m to 10,954 m depth in November 2019 [26]. The method of isolating and culturing fungi from deep-sea sediments was the same as the previous method [31]. The samples and media were prepared in the same way as Peng [26]. The single-spore separation and hyphal-tip purification assays were conducted to obtain a pure culture of the hadal-derived fungi. Each sample was repeated in triplicate. The phenotypes of fungal colonies and pigments were observed and recorded by visual observation every day [46]. The phenotypes of mycelia and conidia were studied using the Nikon DS-Ri2 microscope (Nikon, Tokyo, Japan) and calcium fluorescent white (CFW) dye. The specimen was stained with CFW for one minute, and examined under UV illumination while enlarging it from ×100 to ×400. Unless specific notification, fungal isolates were all cultured on potato dextrose agar (PDA, 20% peeled and sliced potato, 1.0% glucose, and 1.5% agar, with deep-sea seawater, natural pH) at 28 °C and stored at 4 °C.

### 4.2. DNA Extraction, rDNA-ITS Gene Screening and Phylogenetic Analysis

Fungal isolates were identified by combining morphological observations with internal transcription interval (ITS) sequence analysis. Before extracting the total DNA, the hadal-derived fungi were cultured in potato dextrose broth (PDB, 20% peeled and sliced potato and 1.0% glucose, with deep-sea in situ seawater, natural pH) for 7–14 days. Total fungal genomic DNA was extracted using the TIAN combi DNA Lyse & Det PCR Kit (Tiangen Biotech (Beijing) Co., Ltd., Beijing, China) following the manufacturer’s recommendations for fungi. Nearly full-length ITS sequences were amplified by polymerase chain reaction (PCR) using the primers ITS1/ITS4 (5′-TCCGTAGGTGAACCTGCGG-3′/5′-TCCTCCGCTTATTGATATGC-3′) [47]. The PCR mixture (20 μL) was composed of 10 μL 2 × Det PCR Master Mix, 0.5 μL forward primer and 0.5 μL reverse primer, 1 μL DNA template, and 8 μL dd H_2_O. The PCR assay included an initial denaturation step at 95 °C for 3 min, followed by 35 cycles of 30 s at 95 °C, 30 s at 55 °C, 1 min at 72 °C, and then a final extension step of 5 min at 72 °C, before holding at 4 °C.

The amplified ITS sequences were analyzed by GENEWIZ for sequencing and compared within the NCBI (National Centre for Biotechnology Information; http://www.ncbi.nlm.nih.gov) database (accessed on 24 December 2022) for identification. Alignment of the sequences was conducted using Clustal W in the MEGA 7.0 software, conserved motifs were identified, and the sequences were trimmed manually [48]. The unrooted phylogenetic trees of ITS sequences were constructed using the neighbor-joining method, and 1000 replicates were run to calculate bootstrap supports to show the relationship between the isolated strains with the references in the database [49]. More than 98% of similar species were constructed into evolutionary trees to explore the species of the obtained strains.

### 4.3. PKS Gene Screening and Phylogenetic Analysis

The highly conserved sequences of β-ketol synthetase (KS) domains and acyl transferase (AT) domains are shared among PKSs [50]. Several pairs associated with the conserved domains were designed and amplified for fungal PKS genes (Table S5). The pair of primers with the highest amplification efficiency was selected. The PCR mixture (50 μL) consists of 25 μL 2 × Accurate Taq Master Mix (dye plus), 2 μL forward primer and 2 μL reverse primer, 4 μL fungal DNA template, and 17 μL RNase free water. The PCR reaction was as follows: 4 min at 95 °C, 35 cycles of 30 s at 95 °C, 1 min at 50 °C, 2 min at 72 °C, and 7 min at 72 °C. The PCR product was purified with the TIANgel Midi Purification Kit (Tiangen Biotech, Beijing, China) and transformed into a Trans1-T1 phage-resistant chemically competent cell using the pEASY-Blunt Simple Cloning Kit (Transgen Biotech, Beijing, China). The positive recombinants were screened on plates with X-Gal, IPTG (Tiangen Biotech, Beijing, China), and ampicillin by the blue-white screening method following the manufacturer’s instructions. Positive clones were sequenced using vector primers M13F (5′-TAATACGACTCACTATAGGG-3′) and M13R (5′-CAGGAAACAGCTATGACC-3′) by Shanghai Shengong Biotech (Shanghai, China). The obtained PKS gene sequences were analyzed with BlastX at NCBI. Translated protein sequences were obtained from nucleotide sequences using the ORF Finder on the NCBI. The deduced amino acid sequences were used as queries to search the related proteins in the NR protein database using the BLASTP algorithm. An unrooted phylogenetic tree based on amino acid sequences of the KS domain was constructed using the neighbor-joining method in MEGA 7.0 combined with bootstrap analysis with 1000 replications.

### 4.4. Fermentation Culture of Chemical Epigenetic Modifiers of Hadal-Derived Fungi

The chemical epigenetic modifiers 5-azazine (5-Aza) and vorinostat (SAHA) were selected as potential modifiers, with DMSO as a solvent. The agents were filtered by a 0.22 μm filter membrane, and four kinds of culture medium were prepared (Table S6). The spore suspension was prepared prior to the fermentation culture. Fungi were grown on PDA plates for 14 days at 28 °C. Then, the spores were collected by gently scraping the surface of the plates with PDB medium. After mixing and counting with a hemocytometer, the spore suspension was diluted with SDB. Then, 10 μL of spore suspension (above 200 spores/mL) were inoculated on the above medium and cultured at 28 °C for 10 days. Colony phenotypes of fungi were observed and recorded every day.

In order to exclude the influence of manipulation during the preparation of PDA medium on the composition of the medium, Sabouraud dextrose agar (SDA, glucose 4%, peptone 1%, and 1.5–2% agar, with pure water and a natural pH) medium, which was relatively stable in composition, was used as the base fermentation medium. After 14 days of cultivation on SDA medium at 28 °C, the strains were inoculated in Sabouraud glucose broth (SDB, glucose 4%, peptone 1%, pure water, and natural pH) medium containing four concentrations of chemical epigenetic modifiers (0, 50 μM, 500 μM, and 1000 μM) and incubated for 10 days at 28 °C on a rotating shaker (160 rpm). Each group was repeated at least three times.

### 4.5. High Hydrostatic Pressures Culture with Chemical Epigenetic Modifiers of Hadal-Derived Fungi

The spore suspension described above was inoculated into a 10 mL syringe and treated with the stimulated HHP. These syringes were suspended in a pressure vessel filled with pure water and pressurized to 40 MPa at room temperature. The same syringes were incubated under 0.1 MPa at room temperature as the control. Three replicates were maintained for each treatment. After 14 days of incubation, the 100 μL culture medium was re-inoculated on SDA medium and cultured at 28 °C for 10 days under atmospheric pressure.

### 4.6. RNA Extraction and Reverse Transcription

Hadal-derived fungi were cultured in SDB medium for 10 d at 28 °C, and fresh mycelia were harvested. The harvested mycelia treated with different concentrations of 5-Aza were ground into powder in liquid nitrogen and then used for total RNA isolation. Total RNAs of selected fungi were extracted by the RNAiso Plus reagent (TaKaRa, Dalian, China) according to the manufacturer’s instructions. Degradation and contamination of the total RNAs of hadal-derived fungi were detected by 1% agarose gel electrophoresis. The quantity and purity of total RNA were preliminary assessed spectrophotometrically based on A260/A280 and A260/A230 ratios measured by the Nano-Drop 2000 UV–vis spectrophotometer (Thermo Scientific, Wilmington, NC, USA). Then, the primeScript RT reagent kit with gDNA Eraser (Perfect Real Time) (TaKaRa, Dalian, China) was used to synthesize cDNA with 1 µg RNA, according to the recommended instructions. Total cDNA was stored at −80 °C.

### 4.7. Primer Design and Quantitative Reverse Transcriptase PCR (qRT-PCR)

Primer 5 software was used to design the primers for qRT-PCR [51]. To design the degenerate primers, the cDNA sequences of the PKS genes from the homologous species to the isolates were downloaded from NCBI and clustered using Clustal W in the MEGA 7.0 software. The degenerate primers were designed to amplify the PKS gene sequences of our isolates. The amplified sequences were sequenced and the primers were designed for qRT-PCR on Primer 5. The glyceraldehyde-3-phosphate dehydrogenase (GAPDH) gene was employed as the internal reference gene. All qRT-PCR primers (Table S7) and internal reference gene primers (Table S8) in this study were listed in the supplementary.

The qRT-PCR was performed using SYBR Premix Ex Taq II (Tli RNaseH Plus) (TaKaRa, Dalian, China) on the Applied Biosystems 7500 Real-Time PCR System (PerkinElmer Applied Biosystems, Foster City, CA, USA). The PCR mixture (20 µL) contained 10 µL of 2 × SYBR Premix Ex Taq II (Tli RNaseH Plus), 1 µL of cDNA, 0.8 µL of forward and reverse primers, 7 µL of dd H_2_O, and 0.4 µL of 50 × ROX reference dye (TaKaRa, Dalian, China). The PCR reaction was as follows: 30 s at 95 °C, 40 cycles of 5 s at 95 °C, 30 s at 55 °C, and 30 s at 72 °C. Each target gene was amplified using three replicates. Cyclic quantitative (Cq) values were determined based on three biological replicates, each with three technical replicates. The relative quantification of a target molecule relative to the reference gene was performed according to the mean normalized expression (MNE) method [52]. The relative expression was calculated using the 2^−ΔΔCt^ method [53]. All data were analyzed using SPSS Statistics 22. All the results were expressed as the mean ± standard error (SE). The differences between the variables were evaluated by one-way analysis of variance (ANOVA), followed by the least significant difference (LSD) multiple comparison test. Compared with blank, results were considered to be significant at the level of *p* (NS *p* > 0.05, * *p* < 0.05, ** *p* < 0.01, *** *p* < 0.001).

### 4.8. Extraction of Secondary Metabolites of Hadal-Derived Fungi

The extraction of fungal secondary metabolites was performed using the method described in a previous study with minor modifications [54]. After being cultured at 28 °C for 10 days on a rotating shaker (160 rpm), the mycelia and liquids were separated by vacuum filtration with eight layers of sterilized gauze. The secondary metabolites from mycelia and liquids were extracted by ultrasonography with an equal volume of ethyl acetate at room temperature. The extraction was repeated three times. All organic components were collected, mixed, and evaporated to dryness with a 45 °C vacuum rotary evaporator. All crude extracts of the fungal cultures were weighed and stored at −20 °C in the refrigerator.

### 4.9. UPLC-MS/MS Analysis of Secondary Metabolites from Hadal-Derived Fungi

The extracts of hadal-derived fungi were characterized by ultra-performance liquid chromatography/tandem mass spectrometry (UPLC-MS/MS). The crude extracts were dissolved in 1 mL of methanol to the same concentration (100 mg/mL) and filtered by a 0.22 μm filter membrane. UPLC-MS/MS spectrometric analyses were performed using a Vanquish UPLC high-resolution mass spectrometer (Thermo Fisher) equipped with an electrospray ionization (ESI) source operating in the positive ion and negative modes. A Waters ACQUITY UPLC BEH C 18 column (1.7 μm × 2.1 mm × 100 mm, Waters, MA, USA), at a flow rate of 0.4 mL/min, was used for preparative HPLC collection. The column temperature was held at 60 °C, and the injection temperature was held at 10 °C.

### 4.10. Antimicrobial Activity Assay

The Kirby–Bauer method was used to test the antibacterial properties of the crude extracts against pathogens. There were six strains of pathogenic bacteria provided by Shanghai Rainbowfish Company (Shanghai, China), including *Staphylococcus aureus* ATCC25923, *Enterococcus faecalis* FA2-2, *Escherichia coli* MG1655, *Chromobacterium violaceum* ATCC12472 CV026, *Salmonella choleraesuis*, and *Mycobacterium smegmatis*, and three strains of aquatic pathogens, including *Edwardsiel latarda*, *Klebsiella pnenmoniae*, and *Aeromonas hydrophlla*, as the indicated bacteria for this assay. The test sample was quantified at a final concentration of 100 mg/mL with methanol. The indicated pathogens were inoculated in the sterilized Luria–Bertani (LB, 5.0 g yeast extract, 10.0 g tryptophan, 10.0 g sodium chloride, pH adjusted to 7.0) broth medium at 37 °C on a rotary shaker (180 rpm) for 16 h. The culture of indicator pathogens (OD about 0.5) was evenly spread 200 μL of pathogen solution on the LB agar medium. The circular sterile filter papers (6 mm) were placed on the plates. Then, 2 μL of the test sample was dropped onto filter paper, and methanol was used as the negative control. After incubating at 37 °C for 16 h, the inhibition zone diameters were measured and compared with the control to determine the antimicrobial activity. The antibacterial rate was calculated to evaluate the antibacterial effect. All the experiments were repeated three times. All data were analyzed using SPSS Statistics 22.

The MIC of secondary metabolites produced by the isolates was further determined using the micro-broth dilution method as used by Xin et al. [55]. In a 96-well microtiter plate, 100 μL of pathogen suspension was added to each well, and 2 μL of the compound was diluted in DMSO by two-fold serial dilution, then added to each well. Gentamicin was used as a positive control, and DMSO was used as a negative control. Three parallel controls were set up for each experimental well. The 96-well plates were incubated at 37 °C for 24 h.

## Data Availability

The original data presented in the study are included in the article/Supplementary Material; further inquiries can be directed to the corresponding author.

## Supplementary Materials

The following supporting information can be downloaded at: https://www.mdpi.com/article/10.3390/md21110585/s1. All data generated or analyzed during this study are included in this published article (and its Supplementary information files). The datasets supporting the conclusions of this article are available in the NCBI database. The GenBank accession numbers of the ITS genes are listed below: MZ156971 (*A. alternata* CIEL-1), MZ156972 (*Cladosporium* sp. CIEL-2), MZ156974 (*Aspergillus* sp. CIEL-3), MZ156973 (*Arthrinium* sp. CIEL-4), MZ156978 (*S. vesicarium* CIEL-5), MZ156979 (*Alternaria* sp. CIEL-6) MZ156981 (*F. poae* CIEL-7), MZ156980 (*Cladosporium* sp. CIEL-8), OR539202 (*Arthrinium* sp. CIEL-20), OR539207 (*Didymella* sp. CIEL-21), OR539210 (*A. alternata* CIEL-23), OR539237 (*A. alternata* CIEL-24), OR539237 (*A. alternata* CIEL-26), OR539246 (*Preussia* sp. CIEL-27). The GenBank accession numbers of the PKS genes are listed as follows: OR544974 (*A. alternata* CIEL-1), OR544976 (*Cladosporium* sp. CIEL-2), OR544975 (*Aspergillus* sp. CIEL-3), OR544979 (*Arthrinium* sp. CIEL-4), OR544978 (*Alternaria* sp. CIEL-6), OR544977 (*Cladosporium* sp. CIEL-8), OR544984 (*Didymella* sp. CIEL-21), OR544980 (*A. alternata* CIEL-23), OR544981 (*A. alternata* CIEL-24), OR544982 (*A. alternata* CIEL-26), and OR544983 (*Preussia* sp. CIEL-27). Figure S1: Phenotypes of two strains of *A. alternata* isolated and cultured under different epigenetic modifiers; Figure S2: UPLC-MS/MS diagram of secondary metabolites of *A. alternata*; Figure S3: UPLC-MS/MS diagram of secondary metabolites of *A. alternata* under HHP conditions; Table S1: Table of species information based on ITS gene; Table S2: Table of MIC values of fungal secondary metabolites cultured with different epigenetic modifiers for different indicator bacteria; Table S3: Table of species information based on PKS gene; Table S4: Table of the media containing different chemical epigenetic modifiers used in this study; Table S5: List of the qPCR primers involved in this experiment; Table S6: Table of the media containing different concentrations of chemical epigenetic modifiers used in this experiment; Table S7: List of the PKS gene primers involved in this experiment; Table S8: List of the PKS primers involved in this experiment.
