# Supplementary material for: Effects of Epigenetic Modification and High Hydrostatic Pressure on Polyketide Synthase Genes and Secondary Metabolites of Alternaria alternata Derived from the Mariana Trench Sediments
Source: Mar Drugs. 2023 Nov 10;21(11):585. doi: 10.3390/md21110585 (PMC10672368; doi:10.3390/md21110585)
Supplement: Supplementary file 1 [file marinedrugs-21-00585-s001.zip › marinedrugs-2682345-supplementary.pdf]

Figure S1 Phenotypes of two strains of *A. alternata* isolated and cultured under different epigenetic modifiers. PDA was used as the base medium, different epigenetic modifiers were added and cultured at 28 ℃ for 14 days under the same conditions. Each group was repeated three times.

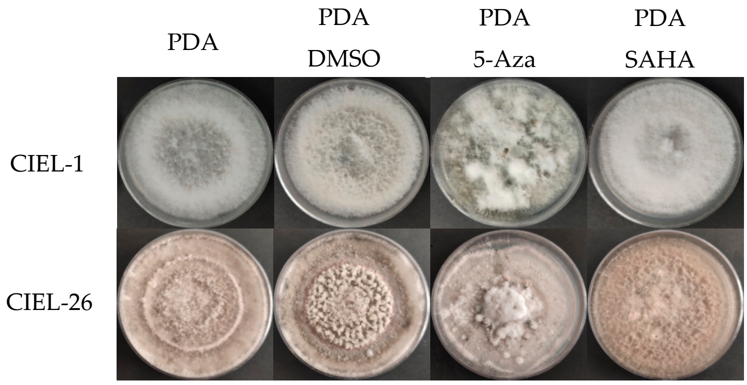

Figure S2 UPLC-MS/MS diagram of secondary metabolites of *A. alternata*. With different concentrations of 5-Aza, two strains of *A. alternata* were cultured under the atmospheric pressure (0.1 MPa) conditions for 14 days and then cultured in SDB at 28 °C for 10 days. In the figure, (**a**) represents *A. alternata* CIEL 1, (**b**) represents *A. alternata* CIEL 26, and (**c**) represents the possible information of the chemical formula. The X-axis was the retention time (min), and the Y-axis was the relative response (%). The signal peaks with no notable alteration in the sample are marked (red). The TIC of the products produced by the target strains in media containing different concentrations of 5-Aza (the number in the upper right corner of the picture indicates the concentration of 5-Aza) was indicated by different colored lines (black-0 μM, red-50 μM, green-1000 μM, and blue-1000 μM).

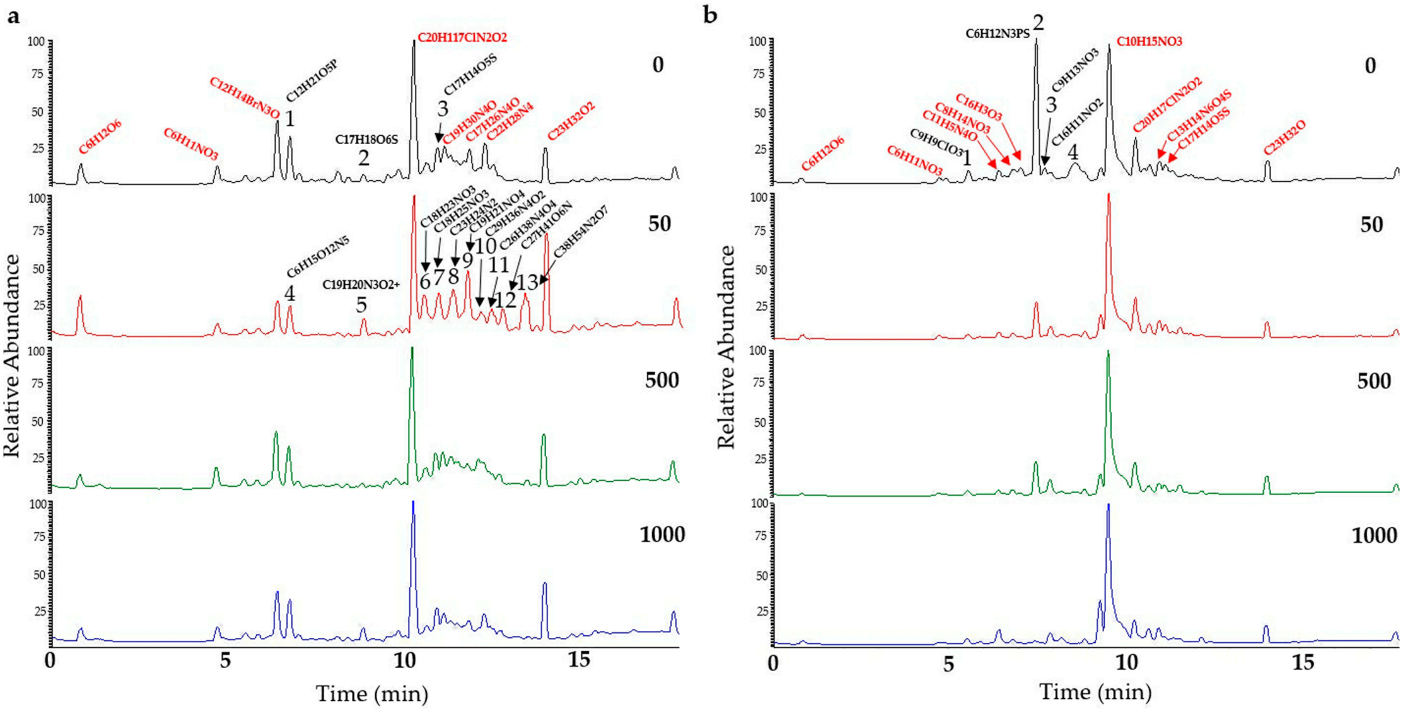

c

No.	Chemical formula	Compound	Structure
**A1-1**	$C_{12}H_{21}O_5P$	Bis(allyloxy)phosphinylacetic acid tert-butyl ester	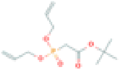
**A1-2**	$C_{17}H_{18}O_6S$	Methyl (1R,4S,5R)-3-(benzothiophen-5-ylmethoxy)-1,4,5-trihydroxy-cyclohex-2-ene-1-carboxylate	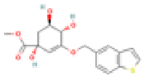
**A1-3**	$C_{17}H_{14}O_5S$	3,4-Dimethyl-2-oxo-2H-1-benzopyran-7-yl benzenesulfonate	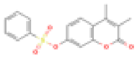
**A1-4**	$C_6H_{15}O_{12}N_5$	Not found	
**A1-5**	$C_{19}H_{20}N_3O_2+$	10-[(2-Hydroxy-2-phenylethyl)amino]-7-methyl-2-aza-7-azoniatricyclo[6.3.1.04,12]dodeca-1(11),2,4(12),7,9-pentaen-11-ol	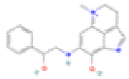
**A1-6**	$C_{18}H_{23}NO_3$	Dobutamine	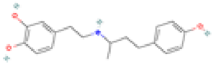
**A1-7**	$C_{18}H_{25}NO_3$	Berkeleyamide A	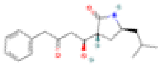
**A1-8**	$C_{23}H_{24}N_2$	1,3-Dibenzyl-2-phenylimidazolidine	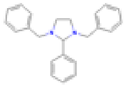
**A1-9**	$C_{19}H_{21}NO_4$	l-Stepholidine	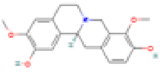
**A1-10**	$C_{29}H_{36}N_4O_2$	4-[2-(4-Methoxyphenyl) phenyl]-N-(3-pyridinylmethyl)-1-piperazinehexanamide	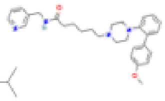
**A1-11**	$C_{26}H_{38}N_4O_4$	Ceanothine C	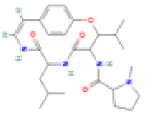
**A1-12**	$C_{27}H_{41}O_6N$	Hydrocortamate	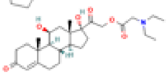
**A1-13**	$C_{38}H_{54}N_2O_7$	tert-butyl N-[6-[4-[[(4E)-4-(7-methoxy-2,2-dimethyl-3H-chromen-4-ylidene)-2,2-dimethyl-3H-chromen-7-yl]oxy]butanoylamino]hexyl]carbamate	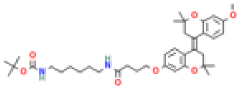
**A26-1**	$C_9H_9ClO_3$	(4-Chloro-2-methylphenoxy)acetic acid	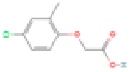
**A26-2**	$C_6H_{12}N_3PS$	Thiotepa	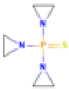
**A26-3**	$C_9H_{13}NO_3$	Epinephrine	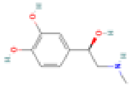
**A26-4**	$C_{16}H_{11}NO_2$	Cinchophen	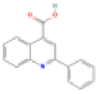

Figure S3 UPLC-MS/MS diagram of secondary metabolites of *A. alternata* under HHP conditions. With different concentrations of 5-Aza, two strains of *A. alternata* were cultured under the HHP (40 MPa) conditions for 14 days and then cultured in SDB at 28 °C for 10 days. In the figure, (**a**) represents *A. alternata* CIEL 1, (**b**) represents *A. alternata* CIEL 26, and (**c**) represents the possible information of the chemical formula. The X-axis was the retention time (min), and the Y-axis was the relative response (%). The signal peaks with no notable alteration in the sample are marked (red). The TIC of the products produced by the target strains in media containing different concentrations of 5-Aza (the number in the upper right corner of the picture indicates the concentration of 5-Aza) was indicated by different colored lines (black-0 μM, red-50 μM, green-1000 μM, and blue-1000 μM).

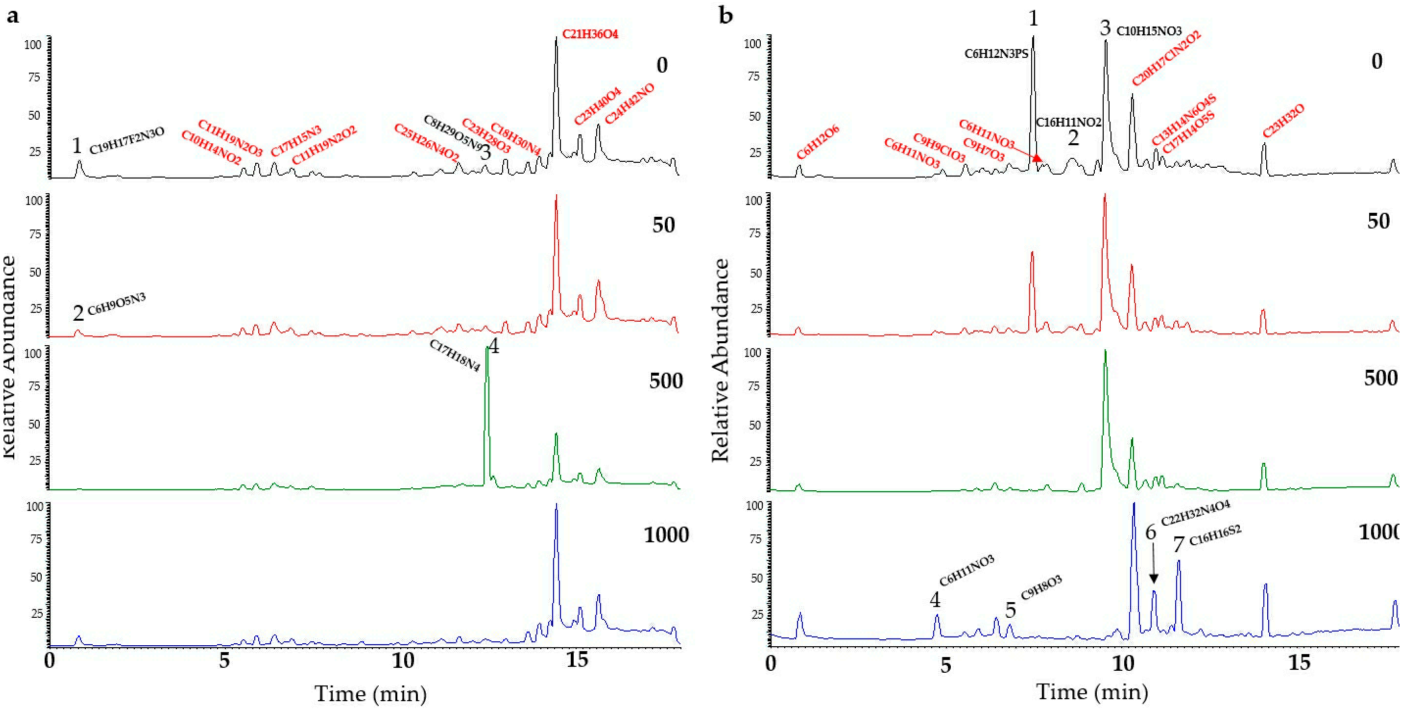

c

No.	Chemical formula	Compound	Structure
**A1-40-1**	$C_{19}H_{17}F_2N_3O$	N-(2,4-difluorophenyl)-1-phenyl-5-propyl-1H-pyrazole-4-carboxamide	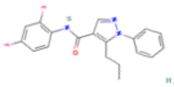
**A1-40-2**	$C_6H_9O_5N_3$	Oxalylalbizziin	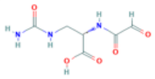
**A1-40-3**	$C_8H_2O_5N_9$	Not found	
**A1-40-4**	$C_{17}H_{18}N_4$	(6,7,8,9-Tetrahydro-5H-pyrimido[4,5-b]indol-4-yl)-p-tolyl-amine	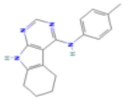
**A26-40-1**	$C_6H_{12}N_3PS$	Thiotepa	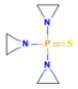
**A26-40-2**	$C_{16}H_{11}NO_2$	Cinchophen	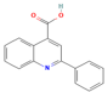
**A26-40-3**	$C_{10}H_{15}NO_3$	Tenuazonic acid	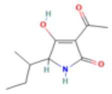
**A26-40-4**	$C_6H_{11}NO_3$	4-Acetamidobutyric acid	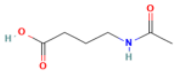
**A26-40-5**	$C_9H_8O_3$	Phenylpyruvic acid	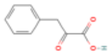
**A26-40-6**	$C_{22}H_{32}N_4O_4$	Tonapofylline	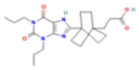
**A26-40-7**	$C_{16}H_{16}S_2$	Cumyl dithiobenzoate	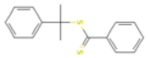

Table S1 Table of species information based on ITS gene. ITS gene sequence results were used as identification information to identify 14 strains of sediment-derived fungi isolated and purified from hadal sediments in the Mariana Trench.

NO.	Name	GenBank accession no.	Fungal genera or species	Similarity (%)	Depth (m)	Sediment Depth (cm)*
1	*Alternaria alternata* CIEL 1	MN822572.1	*Alternaria alternata*	100	5437	0-10
2	*Cladosporium* sp. CIEL 2	MT636978.1	*Cladosporium* sp.	100	7332	0-10
3	*Aspergillus* sp. CIEL 3	MT549144.1	*Aspergillus* sp.	100	5437	10-20
4	*Arthrinium* sp. CIEL 4	KX015984.1	*Arthrinium* sp.	100	6477	0-10
5	*Stemphylium vesicarium* CIEL 5	KY555005.1	*Stemphylium vesicarium*	100	7332	0-10
6	*Alternaria* sp. CIEL 6	KX987252.1	*Alternaria* sp.	100	6477	30-40
7	*Fusarium poae* CIEL 7	DQ297556.1	*Fusarium poae*	100	5437	10-20
8	*Cladosporium* sp. CIEL 8	MN220525.1	*Cladosporium* sp.	100	5437	10-20
9	*Arthrinium* sp. CIEL 20	MH109527.1	*Arthrinium* sp.	100	7332	60-70
10	*Didymella* sp. CIEL 21	MK100198.1	*Didymella* sp.	100	7332	60-70
11	*Alternaria alternata* CIEL 23	MT672690.1	*Alternaria alternata*	100	7332	60-70
12	*Alternaria alternata* CIEL 24	MT672690.1	*Alternaria alternata*	100	7332	60-70
13	*Alternaria alternata* CIEL 26	MT672690.1	*Alternaria alternata*	100	6477	30-40
14	*Preussia* sp. CIEL 27	MK753052.1	*Preussia* sp.	100	6477	30-40

* approximate depth, as these samples were from subsampling of box cores

Table S2 Table of MIC values of fungal secondary metabolites cultured with different epigenetic modifiers for different indicator bacteria. PDA as the base medium, 5-Aza and SAHA as the inhibitors, DMSO as the solvent, and pathogens (*S. aureus, E. faecalis, C. violaceum*, and *S. choleraesuis*) as indicator bacteria. When the crude extract of the metabolite had no antibacterial effect at a concentration of 512 μg/mL, the crude extract of the metabolite was considered to have no antibacterial activity.

S. aureus	1	2	3	4	5	6	7	8	20	21	23	24	26	27
PDA	-	/	256	128	8	128	256	/	-	-	64	128	32	-
PDA+DMSO	-	/	256	32	-	64	-	/	-	256	256	64	-	256
PDA+1 mM 5-Aza	32	/	256	8	-	32	-	/	-	8	128	256	256	-
PDA+1 mM SAHA	128	/	256	256	256	128	-	/	-	256	128	128	-	-

E. faecalis	1	2	3	4	5	6	7	8	20	21	23	24	26	27
PDA	-	/	256	8	-	64	256	/	-	-	32	128	-	-
PDA+DMSO	-	/	256	-	-	64	-	/	-	-	256	64	-	-
PDA+1 mM 5-Aza	8	/	-	128	-	64	-	/	-	128	128	128	256	-
PDA+1 mM SAHA	8	/	256	256	256	64	-	/	-	-	32	128	-	-

C. violaceum	1	2	3	4	5	6	7	8	20	21	23	24	26	27
PDA	8	/	128	128	8	64	128	/	16	64	16	64	16	256
PDA+DMSO	8	/	128	32	8	128	128	/	16	32	32	32	8	64
PDA+1 mM 5-Aza	8	/	128	64	8	64	128	/	32	16	32	64	64	64
PDA+1 mM SAHA	8	/	64	64	64	64	128	/	64	64	16	64	8	/

S. choleraesuis	1	2	3	4	5	6	7	8	20	21	23	24	26	27
PDA	-	/	32	128	32	64	32	/	128	256	32	16	16	16
PDA+DMSO	-	/	32	-	-	16	256	/	32	128	32	8	-	32
PDA+1 mM 5-Aza	64	/	32	256	-	-	256	/	128	16	32	64	32	64
PDA+1 mM SAHA	32	/	32	128	64	64	128	/	32	128	16	64	-	16

Table S3 Table of species information based on PKS gene. PKS sequence results were used as identification information to identify 14 strains of hadal-derived fungi isolated and purified from hadal sediments in the Mariana Trench.

NO.	Name	GenBank accession no.	Fungal genera or species	Similarity (%)	Description
1	*Alternaria alternata* CIEL 1	XP 018388399.1	*Alternaria alternata*	98.58 %	Polyketide synthase PksJ
2	*Cladosporium* sp. CIEL 2	KAF2686893.1	*Lentithecium fiuviatile* CBS 122367	44.40 %	putative PKS
3	*Aspergillus* sp. CIEL 3	XM041705781.1	*Aspergillus puulaauensis*	82.00 %	type I iterative PKS (PKS10)
4	*Arthrinium*sp. CIEL 4	KZL78011.1	*Colletotrichum tofieldiae*	64.44 %	PKS
5	*Stemphylium vesicarium* CIEL 5	-	-	-	-
6	*Alternaria* sp. CIEL 6	KF887238.1	*Alternaria* sp.	97.75 %	putative, PKS gene parial CDS
7	*Fusarium poae* CIEL 7	-	-	-	-
8	*Cladosporium* sp. CIEL 8	PVH82728.1	*Cadophora* sp.	44.73 %	PKS
9	*Arthrinium* sp. CIEL 20	-	-	-	-
10	*Didymella* sp. CIEL 21	KAF3039887. 1	*Didymella heterodera*e	87.39 %	t1PKS
11	*Alternaria lternata* CIEL 23	XP 018388399.1	*Alternaria alternata*	98.58 %	Polyketide synthase PksJ
12	*Alternaria alternata* CIEL 24	XM_018528160.1	*Alternaria alternata*	98.81 %	Polyketide synthase PksJ
13	*Alternaria alternata* CIEL 26	OWY52567.1	*Alternaria alternata*	98.93 %	Polyketide synthase PksJ
14	*Preussia* sp. CIEL 27	XP 002151741.1	*Talaromyces marneffei*	43.94 %	putative PKS

Table S4 Table of the media containing different concentrations of chemical epigenetic modifiers used in this experiment. PDA as the base medium, 5-Aza as the modifier, sterilized water as the solvent, and 0.22 μm filtration membrane were used to filter and remove bacteria.

NO.	Name	Addreviation	Component
1	Sabouraud Dextrose Agar	SDA	glucose 40 g, peptone 10 g, agar 12-15 g, pure water 1 L, natural pH, autoclave (121 ℃, 20 min)
2	Sabouraud Dextrose Agar-50 μM 5-Aza	SDA-50 μM 5-Aza	glucose 40 g, peptone 10 g, agar 12-15 g, pure water 1 L, natural pH, autoclave (121 ℃, 20 min)，50 μM 5-Aza
3	Sabouraud Dextrose Agar-500 μM 5-Aza	SDA-500 μM 5-Aza	glucose 40 g, peptone 10 g, agar 12-15 g, pure water 1 L, natural pH, autoclave (121 ℃, 20 min)，500 μM 5-Aza
4	Sabouraud Dextrose Agar-1000 μM 5-Aza	SDA-1000 μM 5-Aza	glucose 40 g, peptone 10 g, agar 12-15 g, pure water 1 L, natural pH, autoclave (121 ℃, 20 min)，1000 μM 5-Aza

Table S5 List of the PKS gene primers involved in this experiment.

NO.	ID	5'-3'	Product length	Domain
1	LC1F	GATCGTTGGATCCTCTA	17	KS
2	LC2cR	AGATCTCGAGCTCTAGAAT	19	KS
3	GB1	RTRGAYCCNCAGCAICG	17	
4	GB2	GTRCCGTGNCCNTGV	15	
5	KS3	TTYGAYGCIGCITTYTTYAA	20	
6	KS4	RTGRTTIGGCATIGTIATICC	21	KS
7	LC1	GAYCCIMGITTYTTYAAYATG	21	KS
8	LC2	GTICCIGTICCRTGCATYTC	20	
9	LC3	GCIGARCARATGGAYCCICA	20	KS
10	LCS	GTIGAIGTIGCRTGIGCYTC	20	KS
11	KAF1	GARKSICAYGGIACIGGIAC	20	KS
12	KAR1	CCAYTGIGCICCRTGICCIGARAA	24	AT
13	KAF2	GARGCICAYGCIACITCIAC	20	KS
14	KAR2	CCAYTGIGCICCYTGICCIGTRAA	24	AT

Table S6 Table of the media containing different chemical epigenetic modifiers used in this study. PDA as the base medium, 5-Aza and SAHA as the inhibitors, DMSO as the solvent, and 0.22 μm filtration membrane were used to filter and remove bacteria.

NO.	Name	Addreviation	Component
1	Potato Dextrose Agar	PDA	200 g potato (peeled and cut into small pieces), 1.0% glucose, 1.5-2% agar, deep sea in situ seawater, natural pH, Autoclave (121 ℃, 20 min)
2	Potato Dextrose Agar-1 mM DMSO	PDA-1 mM DMSO	200 g potato (peeled and cut into small pieces), 1.0% glucose, 1.5-2% agar, deep sea in situ seawater, natural pH, Autoclave (121 ℃, 20 min), 1 mM DMSO
3	Potato Dextrose Agar-1 mM 5-Aza	PDA-1 mM 5-Aza	200 g potato (peeled and cut into small pieces), 1.0% glucose, 1.5-2% agar, deep sea in situ seawater, natural pH, Autoclave (121 ℃, 20 min)，1 mM 5-Aza
4	Potato Dextrose Agar-1 mM SAHA	PDA-1 mM SAHA	200 g potato (peeled and cut into small pieces), 1.0% glucose, 1.5-2% agar, deep sea in situ seawater, natural pH, Autoclave (121 ℃, 20 min), 1 mM SAHA

Table S7 List of the PKS primers involved in this experiment.

NO.	ID	5'-3'	Seq no	length	GC (%)	TM	Product length
1	AltqpksF1	GAAAGCGTCACCCTGAAGTA	48	20	50	55.2	220
2	AltqpksR1	AAAGGAGGCAGTGGAGCA	267	18	55.6	55.9	220
3	AltqpksR2	AGCCTCTGCACCAAAAGAG	249	19	52.6	55.1	F1/R2=202
4	AltqpksF3	TAAGGAGCGTACACAGGGATT	7	21	47.6	56.8	209
5	AltqpksR3	GTGACCAACATGACCGAGAA	215	20	50	55.9	

Table S8 List of the qPCR primers involved in this experiment.

NO.	ID	5'-3'	Seq no	length	GC (%)	TM	PCR TM	Product length
1	ALTqG1F1	TTGACGGCAACAACCTGA	18	131	50	55.6	54	177
2	ALTqG1R1	TGACGACCTTCTTGGCTC	18	307	55.6	53.3		
3	ALTqG1R2	TTGACACCCATAAC-GAACAT	20	353	40	53.3	54	223
4	ALTqG1R3	GCAGAGGGAG-CAGAAATGA	19	323	52.6	55.6	55	193
5	ALTqG1F4	GGCAAGACCATCCGTTTC	18	157	55.6	55.5	55	169
6	ALTqG1R4	CAGCAGAGGGAG-CAGAAAT	19	325	52.6	55		

ITS sequences

>CIEL-1 *Alternaria alternata*

TTATTGATATGCTTAAGTTCAGCGGGTATCCCTACCTGATCCGAGGTCAAAAGTT-
GAAAAAAAGGCTTAATGGATGCTAGACCTTTGCTGATAGAGAGTGCGACTTGTGCTGCGCTCCGAAACCAGTAGGCCGGC
TGCCAATTACTTTAAGGCGAGTCTCCAGCAAAGCTAGAGACAAGACGCCCAACACCAA-
GCAAAGCTTGAGGGTACAAATGACGCTCGAACAGGCATGCCCTTTGGAATACCAAAGGGCGCAATGTGCGTTCAAAGATT
CGATGATTCACTGAATTCTGCAATTCACACTACTTATCG-
CATTTCGCTGCGTTCTTCATCGATGCCAGAACCAAGAGATCCGTTGTTGAAAGTTGTAATTATTAATTTGTTACTGACGCTG
ATTGCAATTACAAAAGGTTTATGTTTGTCCTAGTGGTGGGCGAACCCACCAAGGAAACAA-
GAAGTACGCAAAAGACAAGGGTGAATAATTCAGCAAGGCTGTAACCCCGAGAGGTTCCAGCCCGCCTTCATATTTGTGTA
ATGATCCCTCCGC

>CIEL-2 *Cladosporium* sp.

GCGGAGGGATCATTACAAGTTGACCCCGGCCCTCGGGCCGGGATGTTCACAACCCTTTGTT-
GTCCGACTCTGTTGCCTCCGGGGCGACCCTGCCTCCGGGCGGGGGCCCCGGGTGGACATTTCAAACTCTTGCGTAACTTTG
CAGTCTGAGTAAATTTAATTAATAAATTAAAACTTTCAACAACGGATCTCTTGGTTCTGG-
CATCGATGAAGAACGCAGCGAAATGCGATAAGTAATGTGAATTGCAGAATTCAGTGAATCATCGAATCTTTGAACGCACA
TTGCGCCCCCTGGTATTCCGGGGGGCATGCCTGTTCGAGCGTCATTTCACCACTCAA-
GCCTCGCTTGGTATTGGGCGACGCGGTCCGCCGCGCGCCTCAAATCGACCGGCTGGGTCTTTCGTCCCCTCAGCGTTGTGG
AAACTATTCGCTAAAGGGTGCCGCGGGAGGCCACGCCGTAAAACAACCCCATTTCTAAGGTT-
GACCTCGGATCAGGTAGGGATACCCGCTGAACTTAAGCATA

>CIEL-3 *Aspergillus* sp.

GTGAATACCTAACACTGTTGCTTCGGCGGGGAACCCCCTCGGGGGCGAGCCGCCGGGGAC-
TACTGAACTTCATGCCTGAGAGTGATGCAGTCTGAGTCTGAATATAAAATCAGTCAAAACTTTCAACAATGGATCTCTTGG
TTCCGGCATCGATGAAGAACGCAGCGAACTGCGATAAGTAATGTGAATTGCAGAATTCAG-
TGAATCATCGAGTCTTTGAACGCACATTGCGCCCCCTGGCATTCCGGGGGGCATGCCTGTCCGAGCGTCATTGCTGCCCAT
CAAGCCCGGCTTGTGTGTTGGGTCGTCGTCCCCCCCGGGGGAC-
GGGCCCGAAAGGCAGCGGCGGCACCGTGTCCGGTCCTCGAGCGTATGGGGCTTTGTCACCCGCTCGACTAGGGCCGGCCG
GGCGCCAGCCGACGTCTCCAACCATTTTTCTTCAGGTTGACCTCGGATCAGGTAGGGATACCCGCTGAACTTAAGCATAT

>CIEL-4 *Arthrinium* sp.

AACCTGCGGAGGGATCATTACAGAGTTATACAACTCCCATACCATCTGTTAACCTACCCAG-
TTATGCCTCGGCGTAAGCTCGGTTGGAGGCACCTGCAGCTACCCTGTAGTTGCGGACTGCCAACTCCAGCCGCGGCCCGCC
GGCGGTACACTAAACTCTGTTTTATTTTATATTCTGAGCGTCTTATTTTAA-
TAAGTTAAAACTTTCAACAACGGATCTCTTGGTTCTGGCATCGATGAAGAACGCAGCGAAATGCGATAAGTAATGTGAAT
TGCAGAATTCAGTGAATCATCGAATCTTTGAACGCACATTGCGCCCATCAG-
TATTCTGGTGGGCATGCCTGTTCGAGCGTCATTTCAACCCTTAAGCCTAGCTTAGTGTTGGGAATCTGCTGTACTGCAGTTC
CTTAAAGACAGTGGCGGAGCGGCGGTAGTCCTCTGAGCGTAGTAATTTATTTCTCGCTTTT-
GTCAGGCTCTGTCCTCCCGCCATAAAACCCCCAATTTTTTAGTGGTTGACCTCGGATCAGGTAGGAATACCCGCTGAACTT
AAGCATATCAATAAGCG

>CIEL-5 *Stemphylium vesicarium*

CAAACACCAAGCAAAGCTGAGGTAACAAATACGCTGAACAGCATGCCCTTTGAATAC-
CAAAGGCGCAATGGCGTTCAAAGATTCATGATTCACGAATTCTGCAATTCACACTACGTATCGCATTTCGCTGCGTTCTTC
ATCGATGCCAGAACCAAGAGATCCGTTGTGAAAGTGTAATAATTACATTGTTTACTGAC-
GCTGATTGCAATCACAAAAAGGTTTATGGTTTGGTCCTGGTGGCGGGCGAACCCGCCCAGGAAACAAGACAGTGCGCAA
AAGACATGGGTGAATAATTCAGACAAGCTGGAGCCCTCAC-
CGAGGTGAGGTCCCAACCCGCTTTCATATTGTGTAAAGAACCCCCTCCGTAGGTGAACCTGCGGAGGGATCATTACACAA
TATGAAAGCGGGTTGGGACCTCACCTCGGTGAGGGCTCCAGCTT-
GTCTGAATTATTCACCCATGTCTTTTGCGCACTTCTTGTTTCCTGGGCGGGTTCGCCCGCCACCAGGACCAAACCATAAACC
TTTTTGTAATTGCAATCAGCGTCAGTAAACAATGTAATTATTACAACTTTCAACAAC-
GGATCTCTTGGTTCTGGCATCGATGAAGAACGCAGCGAAATGCGATACGTAGTGTGAATTGCAGAATTCAGTGAATCATC
GAATCTTTGAACGCACATTGCGCCCTTTGGTATTCCAAAGGG-
CATGCCTGTTCGAGCGTCATTTGTACCCTCAAGCTTTGCTTGGTGTTGGGCGTCTTTGTCTCTCACGAGACTCGCCTTAAAAT
GATTGGCAGCCGACCTACTGGTTTCGGAGCGCAGCACAATTCTTGCACTTTGAATCAGCCTT-
GGTTGAGCATCCATCAAGACCACATTTTCTTAACTTTTGACCTCGGATCAGGTAGGGATACCCGCTGAACTTAAGCATATC
AATAA

>CIEL-6 *Alternaria* sp.
GGGGTAGGAGCTTCCTCCGCTTTTGATATGCTTAAGTTCAGCGGGTATCCCTAC-
CTGATCCGAGGTCAAAAGTTGAAAAAAAGGCTTAATGGATGCTAGACCTTTGCTGATAGAGAGTGCGACTTGTGCTGCGC
TCCGAAACCAGTAGGCCGGCTGCCAATTACTTTAAGGCGAGTCTCCAGCAAAGCTAGAGA-
CAAGACGCCCAACACCAAGCAAAGCTTGAGGGTACAAATGACGCTCGAACAGGCATGCCCTTTGGAATACCAAAGGGCG
CAATGTGCGTTCAAAGATTCGATGATTCACTGAATTCTGCAATTCACACTACTTATCG-
CATTTCGCTGCGTTCTTCATCGATGCCAGAACCAAGAGATCCGTTGTTGAAAGTTGTAATTATTAATTTGTTACTGACGCTG
ATTGCAATTACAAAAGGTTTATGTTTGTCCTAGTGGTGGGCGAACCCACCAAGGAAACAA-
GAAGTACGCAAAAGACAAGGGTGAATAATTCAGCAAGGCTGTAACCCCGAGAGGTTCCAGCCCGCCTTCATATTTGTGTA
ATGATCCCTCCGCAGGCCCCCCTACGGAAAGGAATCCTTACACAAATTTGAAGGCGGGCTG-
GAACCTCTCGGGGTTACAGCCTTGCTGAATTATGCACCCTTGTCTTTTGCGTACTTCTTGTTTCCTTGGTGGGTTCGCCCACC
ACTAGGACAAACATAAACCTTTTGTAGATTGCAATCAGCGTCAGTAACAAATTAA-
TAATTACAACTTTCAACAACGGATCTCTTGGTTCTGGCATCGATGAAGAACGCAGCGAAATGCGATAAGTAGTGTGAATTG
CAGAATTCGGTGAATCATCGAATCTTTGAACGCACATTGCGCACTTTGGTATTCAAAGGG-
CATGACTGTTCGAGCGTCATTTGTACCTAAAGCTTTGCTGGATGATGG

>CIEL-7 *Fusarium poae*
GAAATCTCGGTAAAGTACTTCCGTAGGGGGGACCTGCGGAGGGATCATTACCGAG-
TTTACAACTCCCAAACCCCTGTGAACATACCATATGTTGCCTCGGCGGATCAGCCCGTCCTTCGGGACGGCCCGCCGCAGG
ACCCTAAACTCTGTTTTTAGTGGAACTTCTGAGTAAAAAAACAAA-
TAAATCAAAACTTTCAACAACGGATCTCTTGGTTCTGGCATCGATGAAGAACGCAGCAAAATGCGATAAGTAATGTGAAT
TGCAGAATTCAGTGAATCATCGAATCTTTGAACGCACATTGCGCCCGCCAG-
TATTCTGGCGGGCATGCCTGTTCGAGCGTCATTTCAACCCTCAAGCCCAGCTTGGTGTTGGGAATTGTTTGTCACAGAACAT
TCCCCAAATTGATTGGCGGTCACGTCGAGCTTCCATAGCGTAG-
TAATTTACACATCGTTACTGGTAATCGTCGCGGCCACGCCGTTAAACCCCAACTTCTGAATGTTGACCTCGGATCAGGTAG
GAATACCCGCTGAACTTAAGCATATCAAAAGCCGGAGGAA

>CIEL-8 *Cladosporium* sp.

ACCTGCGGAGGGATCATTACAAGTGACCCCCGGCTCCGGCCGGGGATGTTCATAACCCTTT-
GTTGTCCGACTCTGTTGCCTCCGGGGCGACCCTGCCTTTTCACGGGCGGGGGCCCCGGGTGGACACATCAAAACTCTTGCG
TAACTTTGCAGTCTGAGTAAATTTAATTAATAAATTAAAACTTTCAACAACGGATCTCTT-
GGTTCTGGCATCGATGAAGAACGCAGCGAAATGCGATAAGTAATGTGAATTGCAGAATTCAGTGAATCATCGAATCTTTG
AACGCACATTGCGCCCCCTGGTATTCCGGGGGGCATGCCTGTTCGAGCGTCATTTCACCAC-
TCAAGCCTCGCTTGGTATTGGGCGACGCGGTCCGCCGCGCGCCTCAAATCGACCGGCTGGGTCTTCTGTCCCCTCAGCGTT
GTGGAAACTATTCGCTAAAGGGTGCCACGGGAGGCCAC-
GCCGAAAAACAAACCCATTTCTAAGGTTGACCTCGGATCAGGTAGGGATACCCGCTGAACTTAAGCATATCA

>CIEL-20 *Arthrinium* sp.
AGTTATACAACTCCCACACCATTTGTTAACTTACTCAGTTATGCCTCGGCGTGAACTGCG-
TACGGAGGCAGGTCGGGTGTTACCCTGTAGCCTACCCTGTAGGTTACCCGGTAGCTACCCTGTAGGTTACCCTGTAGCTTA
CCCTGCACCACTCCCGCGCAGCCCGCCGGTGGTACACTAAACTCTT-
GTTTTATTGTATCTTCTGAGCGTATTATTTTAATAATTAAAACTTTCAACAACGGATCTCTTGGTTCTGGCATCGATGAAGAA
CGCAGCGAAATGCGATAAGTAATGTGAATTGCAGAATTCAGTGAATCATCGAATCTTTGAAC-
GCACATTGCGCCCATCAGTATTCTGGTGGGCATGCCTGTTCGAGCGTCATTTCAACCCTTAAGCCTAGCTTAGTGTTGGGAA
TCTACTGTACTGTAGTTCCTTAAAGACAGTGGCGGAGCGATAGTTGTCCTCTGAGCGTAG-
TAAATTTATTTCTCGCTTCTGTAAGGCTCTGTCCTCCCGCCATAAAACCCCCAATTTTTTAGTGGTTGACCTCGGATCAGGTA
GGAATACCCGCTGAACTTAAGCATATCAA

>CIEL-21 *Didymella* sp.
GGAAGGATCATTACCTAGAGTTGTAGGCTTTGCCTGCTATCTCTTACCCATGTCTTTTGAG-
TACCTTCGTTTCCTCGGCGGGTTCGCCCGCCGATTGGACAATTTAAACCATTTGCAGTTGCAATCAGCGTCTGAAAAAATTT
AATAAATTACAACTTTCAACAACGGATCTCTTGGTTCTGGCATCGATGAAGAAC-
GCAGCGAAATGCGATAAGTAGTGTGAATTGCAGAATTCAGTGAATCATCGAATCTTTGAACGCACATTGCGCCCCTTGGTA
TTCCATGGGGCATGCCTGTTCGAGCGTCATTTGTACCTTCAAGCTCTGCTTGGTGTT-
GGGTGTTTGTCTCGCCTCTGCGCGTAGACTCGCCTCAAAACAATTGGCAGCCGGCGTATTGATTTCGGAGCGCAGTACATC
TCGCGCTTTGCACTCATAACGACGACGTCCAAAAGTACATTTTTACACTCTTGACCTCG-
GATCAGGTAGGGATACCCGCTGAACTTAAGCATATCAA

>CIEL-23 *Alternaria alternata*
TGCGGAGGGATCATTACACAAATATGAAGGCGGGCTGGAACCTCTCGGGGTTACAGCCTT-
GCTGAATTATTCACCCTTGTCTTTTGCGTACTTCTTGTTTCCTTGGTGGGTTCGCCCACCACTAGGACAAACATAAACCTTTT
GTAATTGCAATCAGCGTCAGTAACAAATTAATAATTACAACTTTCAACAACGGATCTCTT-
GGTTCTGGCATCGATGAAGAACGCAGCGAAATGCGATAAGTAGTGTGAATTGCAGAATTCAGTGAATCATCGAATCTTTG
AACGCACATTGCGCCCTTTGGTATTCCAAAGGGCATGCCTGTTCGAGCGTCATTT-
GTACCCTCAAGCTTTGCTTGGTGTTGGGCGTCTTGTCTCTAGCTTTGCTGGAGACTCGCCTTAAAGTAATTGGCAGCCGGCC
TACTGGTTTCGGAGCGCAGCACAAGTCGCACTCTCTATCAGCAAAGGTCTAGCATCCATTAA-
GCCTTTTTTTCAACTTTTGACCTCGGATCAGGTAGGGATACCCGCTGAACTTA

>CIEL-24 *Alternaria alternata*
GATCATTACACAAATATGAAGGCGGGCTGGAACCTCTCGGGGTTACAGCCTT-
GCTGAATTATTCACCCTTGTCTTTTGCGTACTTCTTGTTTCCTTGGTGGGTTCGCCCACCACTAGGACAAACATAAACCTTTT

GTAATTGCAATCAGCGTCAGTAACAAATTAATAATTACAACTTTCAACAACGGATCTCTT-
GGTTCTGGCATCGATGAAGAACGCAGCGAAATGCGATAAGTAGTGTGAATTGCAGAATTCAGTGAATCATCGAATCTTTG
AACGCACATTGCGCCCTTTGGTATTCCAAAGGGCATGCCTGTTCGAGCGTCATTT-
GTACCCTCAAGCTTTGCTTGGTGTTGGGCGTCTTGTCTCTAGCTTTGCTGGAGACTCGCCTTAAAGTAATTGGCAGCCGGCC
TACTGGTTTCGGAGCGCAGCACAAGTCGCACTCTCTATCAGCAAAGGTCTAGCATCCATTAA-
GCCTTTTTTTCAACTTTTGACCTCGGATCAGGTAGGGATACCCGCTGAACTTAAG

>CEIL-26 *Alternaria alternata*
CCTGCGGAGGGATCATTACACAAATATGAAGGCGGGCTGGAACCTCTCGGGGTTACAGCCTT-
GCTGAATTATTCACCCTTGTCTTTTGCGTACTTCTTGTTTCCTTGGTGGGTTCGCCCACCACTAGGACAAACATAAACCTTTT
GTAATTGCAATCAGCGTCAGTAACAAATTAATAATTACAACTTTCAACAACGGATCTCTT-
GGTTCTGGCATCGATGAAGAACGCAGCGAAATGCGATAAGTAGTGTGAATTGCAGAATTCAGTGAATCATCGAATCTTTG
AACGCACATTGCGCCCTTTGGTATTCCAAAGGGCATGCCTGTTCGAGCGTCATTT-
GTACCCTCAAGCTTTGCTTGGTGTTGGGCGTCTTGTCTCTAGCTTTGCTGGAGACTCGCCTTAAAGTAATTGGCAGCCGGCC
TACTGGTTTCGGAGCGCAGCACAAGTCGCACTCTCTATCAGCAAAGGTCTAGCATCCATTAA-
GCCTTTTTTTCAACTTTTGACCTCGGATCAGGTAGGGATACCCGCTGAACTTAAGC

>CIEL-27 *Preussia* sp.
CTGCGGAAGGATCATTATCGTGGGGCTTCGGCCCCGTCGAGATAGCACCCTT-
GCCTTTATGAGTACCTTGGTTCCCTCCCCCGTACCTCCGGGGAGCGGGAGGGGCCTCGTCTGTTTCCCCGCGGCGGCGAAA
GCCCCCGGGGACCACGAAACACGCTGTAACCACCTGTAACCGTCTGA-
TAAACAAACAAAAAATCAAAACTTTCAACAACGGATCTCTTGGTTCTGGCATCGATGAAGAACGCAGCGAAATGCGATA
AGTAGTGTGAATTGCAGAATTCAGTGAATCATCGAATCTTTGAACGCACATTGCGCCCTTT-
GGTATTCCTTAGGGCATGCCTGTTCGAGCGTCATTTAAACCTTCAAGCCCTGCTTGGTGTTGGGTGCCTGTCCCGCCCCCGC
GCGTGGACTCACCTCAAATCCATTGGCGGCCCCCGCATGGCCACGAGCGCAGCAGAAAC-
GCAAACTCGTGTGCCGGACCGGGCGGCTCCCAGAAGCTACACTCACCATTTTGACCTCGGATCAGGTAGGGATACCCGCT
GAACTTAAGC

PKS sequences （cDNA）

>CIEL-1 *Alternaria alternata*

GAGGGGCACGGGACGGGGACTCCAGTGGGAGATCCTTGCGAAGCTGCAGCTATTAGCAAC-
GTTTTCTCTTGTCGGACGCCTGAAGACCCGATCTTCGTGGGTGCCCTCAAAAGCAACATGGGTCATCCTGAAGGCGCTAGT
GGAATCGCGGGTGTGATTAAGACGCTTCTTGTTCTAGAGAAGGGCATCATTCCAC-
CGAATGTATATCCTGAGCGCATCAGCCCGGCTGTTGCGGCGGCTGGCCCCGACTTGAAGTTTCCGCTCGTACCGGCAACCT
GGCCGACGGATGGTATTCGACGCGCGAGTGTGAACTCATTCGGGTATGGAGGCACGAAC-
GCCCACGTTGTTCTGGACGATGCGCTGAGCTTTCTTCGTGATCATGGTTTGTCCGGCCAACATTGCACTGAAGTGCTTGACG
GTAGCAAAGGAGCAGCTGAACCAGCCACCTACGATGGCCTAGCGATCAATAGTGAGGACGG-
CAGCTATAGCACGGGTACTAACGCATATTCGCAATCCTTTGCAGACGATACGCCACCTGATATCATGGACGACTACGAAA
CCGCCCCAAAGCTGTTCGTACTATCCGCATTTGAC-
GAGCGTGCCGTCCAGCGATCTATCCCCACTTTCGAAAAGTGGTTGTGCAATCATGCGAACGATGAGAATGATCACCGGAT
TCTTAATGATGTAGCGTACACCTTGGCTGAGAAGCGAACGTCATTTCCATGGAAGACTGCTT-
GCGTTGCTTTGCCAAATCTGCTCTCGCAACTCTCCTGGTCTACCCCTACGCGAGTCAAACAGCGAGTGAATCTGTGTTTCGT
CTTCTCCGGCCACGGCGCCCAATGG

>CIEL-2 *Cladosporium* sp.

GAAGGGCATGGGACGGGGACAGTGGCAGGAGACGCAGCCGAGCTAGGTGCCATT-
GGCGATGCATTCGGGCCATCGCGTCCGATTGACCAACCCTTACTTGTTGGCAGCGTCAAAACAAACGTCGGTCACTTAGA
AGGCACCGCCTCTCTAGCAGGCATCATCAAGGCCATTTTAAGCGTGGA-
GAAAGGTGTCATTCCGCGGAATCTGAACTTTGAGCGACCAAATCCGAACAACGACCTTGAAAAATACCGCATTTCTGTTC
CGACTCAGTTGATGCAATGGCCCTTGCATGGTGTGAGAAGGGCTAGTGTCAATTGTTTCG-
GATTCGGCGGAACCATTGCTCACGTGATCCTGGACGACATTGCCAGTTTTGCGAGTGAGAACAAGCTGTCTGCACGTCACG
TCACAGTGTCCCCGTTCGAGAGCCACCTCAATGGACATTCAAATATCGATA-
CATCAATGTCCGGACATGTCGAGTCCGGGAAGAATTCTCTGGCACTGCCTTATCGTTTGCTGGCCCTTTCGGCACCTGAGC
AAGACGGTGTACAGCGGAATGCGGAGATACTGGCGAAGCATCTTTCAAAGATCTCGTT-
GCCATCTGACAGCTTCCAGGGCAACTCGCCATTGGACGATTTCTGCTACACACTAAACCTCCGCAGAACGCATTTCCAATG
GAGGAGTATCGCAGTGGATCAATCTCAGCTGGGGATGAGCCACTGCCTTTCCAC-
TCTCCCTCGGGCTAACAAAATCATCGAGGACTGCAAAGTGTGCTTCGTCTTCTCCGGCCATGGCGCCCAATGG

> CIEL-3 *Aspergillus* sp.

GAGGGGCACGGGACGGGGACGCCGGTTGGAGATCCTGTGGAGGCGAGGGCTATCAG-
TGAGGCATTTATCAATTTTCGTCTTCTTCTTCTTCTACGCCGGACAGGTCAGATACAATCCACGTCGGGTCTATCAAAACCG
TCATCGGGCATTTAGAGGGCTGTGCGGGATTAGCGGGCGTGCTGAAGGCCATACAGGC-
TATTAAGCACAAGATCATCCCTCCAAACCTCCTCTTCAACGAACTGAATCCAGAAATTGAACCGTATTATGGGCCGTTACA
GATCACGAAAGAACCACTCAGTTGGCCAGAACGGCCAGCAGGTTCAC-
CGATGCGCGCGAGCGTGAATAGTTTCGGATTCGGGGGAACCAATGCGCACGCGATTATCGAGAGTTTTGCAGAAGAGAGC
AACATTAACATTGCGAAAGAACATGTCCCTGAGGAAGAGTTGAG-
TCCGGCTGTCCCGTTATTCTACTCAGCTCGGTCTGGGTCGTCGTTGCTGCGTACCATCAAAGCATATATCCAGCATCTGCGG
CATGACAACCCGTGTATAGATCTTCGCGATCTCAGCTGGATTCTCTACTCGCGCCGCTCCAC-
GCATCGAATACGAGCTTCTTTTTCTGGAGTATCTCGAGATGCCATCTTGGAGAAAATGGAACGCTACGTCTCGCGCCGTGA
GGCTGGATACGAACCACCACTGATGAATCAGAGCCCTAGAATTCTAGGAATATTCTCCGGCCACGGCGCCCAGTGG

>CIEL-4 *Arthrinium* sp.
GAATGGCACGGGACGGGGACTCCTATCGGCGATCCTCTCGAAGCGAAGGCCCTGGGAC-
GTACTTTTGGAAATGCAAGACAGAGCGGCGATTCCGTATATATCGGATCTCTGAAGTCCAATATAGGGCATCTGGAAGGT
GGATCTGGCACAGCCCAGGTGATCAAAGCAATCTTCATGCTGGAG-
CAGGGACAAATTCCCCCTTCCCTCTACTACGAAAAGCCCAATCCCCATATCCCGATGGACGACTGGAACCTCCGAGTACC
TACAGAACTCACTCCCTGGCCTGCCGATGGCCTCCGTCGTATTAGCATCAACTCTTTTGGC-
TATGGAGGTACGAACGCTCACTGCATTCTTGATGATGCTTACCACTACCTTAAGGAGAGACGACTAGCTGGAAATCACAA
TGTGAAAGTCGCAGACGGGTCGTCCCCTGCGATCTCTGAA-
GACTCAGGGGTGTCTCTCAGCGAGCCGATTGGCCCCCTTACGCTAAGAAATACTGATACTGAGAGCGAGACGGAGCCCGA
CAAAGCAATGGCGCTATTTCCTCGTCTTCTGATTTGGAGCTCCAATGAGAAGGAAGGCG-
TATACCGAACGTGTGCTGCTCATGCATCCTATCTCAAATCGAAGTTTGCTGAGCTGGAGGCCAAGCATAAATCGGAAGTTT
TCAGCAAACTTATACGCACACTTCATGCTCGACGAAGCCGATTGCCGTG-
GAAGTCCTTCTCGATCGTCAATTCTCTTAATGTGGTCGGGACTCTCGAGGGTTCAATTGTGGAACCGGTGCGTTCGAGTGGC
GAGAAACCACCTCTAATCTTCATCTTTTCCGGCCACGGCGCCCAGTGG

>CIEL-6 *Alternaria* sp.
GAGGCGCATGGGACGGGGACTCCAGTGGGAGATCCTTGCGAAGCTGCAGCTATTAGGCAAC-
GTTTTCTCTTGTCGGACGCCTGAAGACCCGATCTTCGTGGGTGCCCTCAAAAAGCAACATGGGTCATCCTGAAGGCGCTAG
TGGAATCGCGGGTGTGATTAAGACGCTTCTTGTTCTAGAGAAGGGCATCATTCCAC-
CGAATGTATATCCTGAGCGCATCAGCCCGGCTGTTGCGGCGGCTGGCCCCAACTTGAAGTTTCCGCTCGTACCGGCAACCT
GGCCGACGGATGGTATTCGACGCGCGAGTGTGAACTCATTCGGGTATGGAGGCACGAAC-
GCCCACGTTGTTCTGGACGATGCGCTGAGCTTTCTTCGTGATCATGGTTTGTCCGGCCAACATTGCACTGAAGTGCTTGACG
GTAGCAAAGGAGCAGCTGAACCAGCCACCTACGATGGCCTAGCGATCAATAGTGAGGACGG-
CAGCTATAGCACGGGTACTAACGCATATTCGCAATCCTTTGCAGACGATACGCCACCTGATATCATGGACGACTACGAAA
CCGCCCCAAAGCTGTTCGTACTATCCGCATTTGAC-
GAGCGTGCCGTCCAGCGATCTATCCCCACTTTCGAAAAGTGGTCGTGCAATCATGCGAACGATGAGAATGATCACCGGAT
TCTTAATGATGTAGCGTACACCTTGGCTGAGAAGCGAACGTCATTTCCATGGAAGACTGCTT-
GCGTTGCTTTGCCAAATCTGCTCTCGCAACTCTCCTGGTCTACCCCTACGCGAGTCAAACAGCGAGTGAATCTGTGTTTCGT
CTTCTCCGGCCACGGCGCCCAATGG

>CIEL-8 *Cladosporium* sp.
GAAGGGCACGGGACGGGGACTGTTGCGGGAGATGCCGCAGAGCTCGGCGCGATCGGAGAC-
GCCTTTGGTGCAACACGACTTTCCGACCAGCCTTTGAATGTGGGAAGCGTGAAGACCAACGTTGGCCATCTCGAGGGAAC
AGCTTCCCTGGCCGGCATCATCAGGGCTGTCCTCAGCTTGGAAAAGGGTGTCAT-
ACCCCAGAACTTGAATTTCGAGCGACCGAATCCGAAGAACGATCTCGAAAAGTATAGGATTACCGTTCCGACTAATCTAA
CGAAGTGGCCTTTACAAGGAGTCAGGAGAGCCAGCATCAACTGCTTTGGATTCGGTGGAAC-
GATCGCTCATGTCATCTTGGATGATGCGGAGAGCTTTTTGACCGAGCGAATGCTATTTGCGAATCATGTTACGGGCTCTCCG
ATCTCAAACGGCGGACTAAGTGGCATCATGGTAGATGACAGCACCAATTCGCGATCCG-
CATTTCTGCCATACCGTTTACTCGCATTGTCTTCACCCGAGCAGGATGGCGTCAAACGAAATGCGAACGCACTAGCCGATT
ACAATGCTACGAAGACCGATTCGAATGGAGACACGGGAAATTCATATCTAGACGATCTCTGC-
TACACGTTCAACCTTCGCAGAACGCAATTTCCATGGAGACAAGCCGTTGCTGTACAGACAGCAACCGACGTCAGCGAGCA
GCTTGACGTCCTACCCAAGGCTTCGAAGGCCATCGAGGACTGCAGGGCTT-
GCTTCGTCTTCTCCGGCCATGGCGCCCAGTGG

>CIEL-21 *Didymella* sp.
GAGTGGCATGGGACGGGGACCCGTGTTGGTGACCCAATTGAGATTGGTGCGATTCAAAGAG-
TTTTCGGAGACGGGAGGACCAAACGCAAGCCCCTCTATATCGGGTCTGTCAAATCTAACATTGGTCACCTGGAAGCAGCG
GCTGGTAAGTTCTGCACTAATTGAGCTTCTCGAGAGCCTCGAGAAGCCCTCG-
CATGTGTTTCTCGAGCTAACGTTTTCAGGCATCGCAGGAGTGATCAAGACTGCGCTAATGTTGGAGCGTGGATTTATCCTCC
CAAACTATGACTTCAAATATCCGAACGAAAATATCCCCTTTGACCAGTGGGGCCTAAAGGTT-
GCGACTCGTCAACAGCCCTGGCCTTTTGGCAAGCTTTGGGCCAGTGTCAATGGCTTTGGGTTTGGAGGAACTAACGGACAT
GTTGTGGCAAGTTTCCCAGTTCTATTCTATTGTTGAAGTTGCGTATGCTAACCGCCGCTTTT-
GTCGTATAGATGACTAGAGGTCCATTGGAACGTAAGACGATGAAGGAAGAAGTTGACACCCAAACTTGCGAGCGTTTGTT
CATTCTATCCGCGAACGATAAATCGAGTGCCGAGAAGACGATGCAAAACCTTGG-
TATCTATCTCGAGCAGCGTCCAGAGATCTTTCAGAACGGTCTCTTGAGCAATCTCGCTTACACATTGGGACAGCGGAAGTC
TGTTCATCCATGGCGCATTGCTGTATCCGCGTCCTCCAGTGCAGAGTTGGTCGAAACCTT-
GTCTAGTGGTAGGATTAGTCCCATCAAGCAAGACGCTGATACACCACGTTTGGCATGGATCTTCTCCGGCCATGGCGCCCA
ATGG

>CIEL-23 *Alternaria alternata*
GAGGGGCATGGGACGGGGACTCCAGTGGGAGATCCTTGCGAAGCTGCAGCTATTAGCAAC-
GTTTTCTCTTGTCGGACGCCTGAAGACCTGATCTTCGTGGGTGCCCTCAAAAGCAACATGGGTCATCCTGAAGGCGCTAGT
GGAATCGCGGGTGTGATTAAGACGCTTCTTGTTCTAGAGAAGGGCATCATTCCAC-
CGAATGTATATCCTGAGCGCATCAGCCCGGCTGTTGCGGCGGCTGGCCCCAACTTGAAGTTTCCGCTCGTACCGGCAACCT
GGCCGACGGATGGTATTCGACGCGCGAGTGTGAACTCATTCGGGTATGGAGGCACGAAC-
GCCCACGTTGTTCTGGACGATGCGCTGAGCTTTCTTCGTGATCATGGTTTGTCCGGCCAACATTGCACTGAAGTGCTTGACG
GTAGCAAAGGAGCAGCTGAACCAGCCACCTACGATGGCCTAGCGATCAATAGTGAGGACGG-
CAGCTATAGCACGGGTACTAACGCATATTCGCAATCCTTTGCAGACGATACGCCACCTGATATCATGGACGGCTACGAAA
CCGCCCCAAAGCTGTTCGTACTATCCGCATTTGAC-
GAGCGTGCCGTCCAGCGATCTATCCCCACTTTCGAAAAGTGGTTGTGCAATCATGCGAACGATGAGAATGATCACCGGAT
TCTTAATGATGTAGCGTACACCTTGGCTGAGAAGCGAACGTCATTTCCATGGAAGACTGCTT-
GCGTTGCTTTGCCAAATCTGCTCTCGCAACTCTCCTGGTCTACCCCTACGCGAGTCAAACAGCGAGTGAATCTGTGTTTCGT
CTTCTCCGGCCATGGCGCCCAATGG

>CIEL-24 *Alternaria alternata*
CCATTGGGCGCCGTGGCCGGAGAAGACGAAACACAGATTCACTCGCTGTTTGACTCGCG-
TAGGGGTAGACCAGGAGAGTTGCGAGAGCAGATTTGGCAAAGCAACGCAAGCAGTCTTCCATGGAAATGACGTTCGCTTC
TCAGCCAAGGTGTACGCTACATCATTAAGAATCCGGTGATCATTCTCATCGTTCG-
CATGATTGCACAACCACTTTTCGAAAGTGGGGATAGATCGCTGGACGGCACGCTCGTCAAATGCGGATAGTACGAACAGC
TTTGGGGCGGTTTCGTAGTCGTCCATGATATCAGGTGGCGTATCGTCTG-
CAAAGGATTGCGAATATGCGTTAGTACCCGTGCTATAGCTGCCGTCCTCACTATTGATCGCTAGGCCATCGTAGGTGGCTG
GTTCAGCTGCTCCTTTGCTACCGTCAAGCACTTCAGTGCAATGTTGGCCGGACAAAC-
CATGATCACGAAGAAAGCTCAGCGCATCGTCCAGAACAACGTGGGCGTTCGTGCCTCCATACCCGAATGAGTTCACACTC
GCGCGTCGAATACCATCCGTCGGCCAGGTTGCCGGTACGAGCGGAAACTTCAAGTT-
GGGGCCAGCCGCCGCAACAGCCGGGCTGATGCGCTCAGGATATACATTCGGTGGAATGATGCCCTTCTCTAGAACAAGAA
GCGTCTTAATCACACCCGCGATTCCACTAGCGCCTTCAGGATGACCCATGTTGCTTTT-
GAGGGCACCCACGAAGATCGGGTCTTCAGGCGTCCGACAAGAGAAAACGTTGCTAATAGCTGCAGCTTCGCAAGGATCTC
CCACTGGAGTCCCCGTCCCGTGCGCCTC

>CIEL-26 *Alternaria alternata*

GAGTGGCATGGGACGGGGACTCCAGTGGGAGATCCTTGCGAAGCTGCAGCTATCAG-
CAATGTTTTCTCTTGTCGGACGCCCGAAGACCCGATCTTCGTGGGTGCCCTCAAAAGCAACATGGGTCATCCTGAAGGCGC
TAGTGGAATCGCGGGTGTGATTAAGACGCTTCTTGTTCTAGAGAAGGGCATCATTCCAC-
CGAATGTATATCCTGAGCGCATCAGCCCGGCTGTTGCGGCGGCTGGTCCCAACTTGAAGTTTCCGCTCGTACCGGCAACCT
GGCCGACGGATGGTATTCGACGCGCGAGTGTGAACTCATTCGGGTATGGAGGCACGAAC-
GCCCACGTCGTTCTGGACGATGCGCTGAGCTTTCTTCGTGATCATGGTTTGTCCGGCCAACATTGCACTGAAGTGCTTGACG
GTAGCAAAGGAACAGCTGAACCAGCTACCTACGATGGCTTAGCGATCAATAGTGATGAC-
GACAGCTATAGCACGGGTACTAACGCATGTTCGCAAACCTTTGCAGACGATACGCCACCTGATATCATGGACGACTACGA
AACCGCCCCAAAGCTGTTCGTACTATCCGCATTTGAC-
GAGCGTGCCGTCCAGCGATCTATCTCCACTTTCGAAAAGTGGCTGCGCAATCATGCGAACGATGAGAATGATCACCGGAT
TCTTAATGATGTAGCGTACACCTTGGCTGAGAAGCGAACGTCATTTCCATGGAAGACTGCTT-
GCGTTGCTTTGCCAAATCTGCTCTCGCAACTCTCCTGGTATACCCCTACGCGAGCCAAACAGCGAGTGAATCTGTGTTTCGT
CTTCTCCGGCCACGGCGCCCAGTGG

>CIEL-27 *Preussia* sp.

GGGCATGGGACGGGGACGCAGGCTGGTACGTCGTTTACCCTTGGCG-
CAGAAAATTTTTCTGACTCACCCTGACTAGGTGACAACGCCGAAATCAACTCGATTTCGGAGGTATTTTGCGGCCAAGGAC
GTGAACGGGATCTTTATGTAGGCTCAGTCAAAGCCAACATCGGCCACTTGGAAGCCG-
CAAGTGGCGTGGCTGGACTGATCAAGGTGGTTATGATGCTCAAGAAGGACCAAATACCGCCTCATATTGATCTTGTAGAA
CCCAAGCCAACACTTAGGCTTGAAGAGAGAGGAATAAAGGTAAGCATGTCTCCTCGCAG-
TTCAATGCTGTTTTCGTTTACTAATGTTTTGAGCTAGATCGCGCAGGAACCCACGAGTCTGACTCCGAGTGACCATATAGGT
CCTCGCAGGGTATCACTGAACTCATTCGGATATGGCGGTACTA-
ACTGTCATCTCATTCTCGAGGGATATGATGCGTCGCAGCCTAAAGCAACGTCTTCGAATGGCGAGATTTCTGAAGGTTGGC
TTTCCACGGAAACCGCTTCTGAACCACTGCTCCCGTTTCCACTGACCGCTGCTTCTGA-
GACTGCCCTTCAGGCAACTTGCAAGCGCCTTAGCCAATGGATTGTGGACACGAGACCGTCTGAATCCGAACTCCGCGATC
TTGCGCACACACTTGGAAGTCGCCGCTCCCACTTACCATGTCGAA-
GAACTGTTCTGGCTTCCACGATCGAGGAACTACAGGCAGAGCTCACAGCCGAAAAAGCACTAGTTGTCAAGGCCGGCTCG
TCTCCGAAAATGACCATGGCTTTTTCCGGCCATGGCGCCCAGTGGA
